# Free-electron crystals for enhanced X-ray radiation

**DOI:** 10.1038/s41377-023-01363-4

**Published:** 2024-01-24

**Authors:** Lee Wei Wesley Wong, Xihang Shi, Aviv Karnieli, Jeremy Lim, Suraj Kumar, Sergio Carbajo, Ido Kaminer, Liang Jie Wong

**Affiliations:** 1 School of Electrical and Electronic Engineering, Nanyang Technological University, 50 Nanyang Avenue, Singapore, 639798 Singapore; 2 Solid State Institute and Faculty of Electrical and Computer Engineering, Technion – Israel Institute of Technology, Haifa, 3200003 Israel; 3 School of Electrical Engineering, Fleischman Faculty of Engineering, Tel Aviv University, Tel Aviv, 69978 Israel; 4 Department of Applied Physics, Stanford University, Stanford, CA 94305 USA; 5 Science, Mathematics and Technology, Singapore University of Technology and Design, 8 Somapah Road, Singapore, 487372 Singapore; 6 Electrical and Computer Engineering Department, UCLA, 420 Westwood, Los Angeles, CA 90095 USA; 7 Physics and Astronomy Department, UCLA, 475 Portola Plaza, Los Angeles, CA 90095 USA; 8 SLAC National Accelerator Laboratory, Stanford University, 2575 Sand Hill Road, Menlo Park, CA 94025 USA

**Keywords:** Quantum optics, Single photons and quantum effects, Nanophotonics and plasmonics, X-rays

## Abstract

Bremsstrahlung—the spontaneous emission of broadband radiation from free electrons that are deflected by atomic nuclei—contributes to the majority of X-rays emitted from X-ray tubes and used in applications ranging from medical imaging to semiconductor chip inspection. Here, we show that the bremsstrahlung intensity can be enhanced significantly—by more than three orders of magnitude—through shaping the electron wavefunction to periodically overlap with atoms in crystalline materials. Furthermore, we show how to shape the bremsstrahlung X-ray emission pattern into arbitrary angular emission profiles for purposes such as unidirectionality and multi-directionality. Importantly, we find that these enhancements and shaped emission profiles cannot be attributed solely to the spatial overlap between the electron probability distribution and the atomic centers, as predicted by the paraxial and non-recoil theory for free electron light emission. Our work highlights an unprecedented regime of free electron light emission where electron waveshaping provides multi-dimensional control over practical radiation processes like bremsstrahlung. Our results pave the way towards greater versatility in table-top X-ray sources and improved fundamental understanding of quantum electron-light interactions.

## Abstract (web summary)

Shaping the quantum free electron wavefunction leads to over thousand-fold intensity enhancements and greater directionality in X-ray bremsstrahlung.

## Graphical abstract

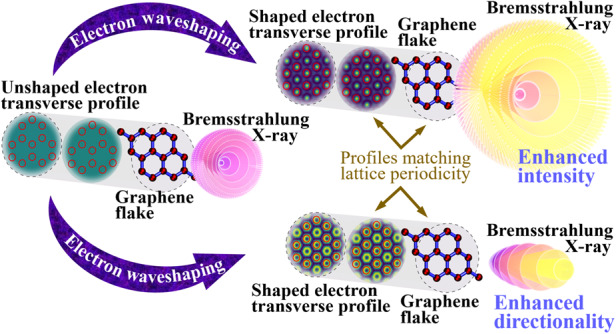

## Introduction

Spontaneous light emission driven by free electrons is one of the most fundamental interactions in quantum electrodynamics (QED). Serving as the cornerstone for a wide range of tunable light sources, these emission processes come in various forms and span many octaves from the THz regime to the X-ray regime, e.g., bremsstrahlung^1,2^, Cherenkov radiation^3–10^, transition radiation^11,12^, inverse Compton scattering^13–15^, Smith–Purcell radiation^8,16–25^, undulator^2,26–28^, and synchrotron radiation^29^.

Bremsstrahlung, one of the most prevalent free electron spontaneous emission processes observed and harnessed in science and industry today, involves scattering of free electrons off the atomic nucleus, and emitting photons from the resulting electron deceleration. Approximately 80% of the X-ray emission in modern X-ray tubes come from bremsstrahlung, which is characterized by a continuous spectrum and a broad angular distribution^30–32^. X-rays are indispensable to modern science through X-ray spectroscopy^33,34^, X-ray crystallography^35–37^; and also indispensable to medicine and industry through radiography^38^, radiotherapy^39,40^, X-ray computed tomography^41^, and materials characterization^42^. Therefore, discovery of efficient mechanisms for intense, versatile bremsstrahlung emission is potentially transformative to today’s scientific, medical, and industrial landscape.

To this end, emerging phenomena arising from the quantum wave nature of free electrons^2,24,43,44^ prompt one to consider whether the technologies developed to shape the electron wavefunctions could be used to enhance bremsstrahlung. For example, the electron pulse spatio-temporal profile can be reduced to attosecond timescale via mechanisms such as static field^45–50^, laser pulses^51–66^, surface plasmon polaritons (SPPs)^67^, radio-frequency cavities^68–73^ and material structure^74,75^. Other popular shaping methods include amplitude masks^76^, phase masks, and even programmable phase plates^77–83^. Research shows that electron propagation trajectory and intrinsic properties like orbital angular momentum (OAM)^5,84,85^ and spin angular momentum^86,87^ can be manipulated via varying the electron phase structure using amplitude and phase holograms^79,88–91^, etc.

The free electron waveshaping for controlling various types of QED processes has been widely studied, ranging from the enhancement of free-electron-bound-electron resonant interaction^92,93^, the modification of bremsstrahlung emission’s spatial and spectral characteristics^2^, the quantum interference between different QED processes^94^, to the creation of optical cat states and Gottesman–Kitaev–Preskill (GKP) states^95^. Meanwhile, studies on free electron X-ray emission from nanomaterials and metamaterials (typical two-dimensional crystals, e.g., graphene, van der Waals materials) provide another important aspect on controlling X-ray generation^44,96–100^. However, scaling up bremsstrahlung X-rays via electron waveshaping remains an open question.

Here, we show from foundational QED theory that highly intense bremsstrahlung X-ray emission from a single electron can indeed be achieved by shaping its wavefunction. In particular, this enhancement is achieved when the free electron wavefunction is shaped such that its spatial probability profile contains periodic patterns matching the crystal lattice. We show that the bremsstrahlung intensity scales linearly as the number of atoms ${N}_{{\rm{a}}}$ (or more specifically, the number of lattice unit cells) inside the electron wavefunction’s transverse coherent cross section in the crystalline scatterer. Furthermore, the bremsstrahlung intensity also scales linearly with the number of electron eigenstates ${N}_{{\rm{s}}}$ comprising the shaped electron wavefunction. The resulting enhancement even shows quadratic scaling of ${N}_{{\rm{s}}}$ in cases where a certain type of output emission profile is considered. Combining both ${N}_{{\rm{a}}}$ scaling (from crystalline scatterer) and ${N}_{{\rm{s}}}$ scaling (from shaped electron wavefunction), one can achieve more than three orders of magnitude enhancement for bremsstrahlung in the hard X-ray regime.

Our findings also address the fundamental question as to whether the intensity of bremsstrahlung can be fully attributed to the improved overlap between the electron spatial probability distribution and the atomic centers. Our investigation shows that the resulting output emission angular profile cannot be fully explained in such a simple way based on existing paraxial and non-recoil theory for free electron light emission. Instead, the recoil corrections together with the strong nonparaxiality of the incident electron wavefunction result in emission patterns that are sensitive to the electron phase.

## Results

To describe scattering processes such as bremsstrahlung using QED, we consider an arbitrary electron initial state $\left|{i}_{1}\right\rangle$ and the vacuum state for the electromagnetic field, $\left|0\right\rangle$, i.e., $\left|{i}_{1},0\right\rangle$. The initial joint state of the electron and the field is transformed via a scattering operator, in the form of a scattering matrix $\hat{{\rm{S}}}$, into an output state comprising a final electron state $\left|f\right\rangle$ and a single photon state $\left|{1}_{{\bf{k}}}\right\rangle$ with mode ${\bf{k}}$, i.e.,$\left|f,{1}_{{\bf{k}}}\right\rangle$. The transition probability given by ${\left|{M}_{1}\right|}^{2}$, where ${M}_{1}=\left\langle f,{1}_{{\bf{k}}}\right|\hat{{\rm{S}}}\left|{i}_{1},0\right\rangle$, represents how frequently a process can happen, and captures the intensity of the photon emission in the bremsstrahlung process. By means of shaping the electron’s initial state, e.g., through the superposition of two individual states $\left|{i}_{1}+{i}_{2},0\right\rangle$, the transition probability takes the form ${\left|\left\langle f,{1}_{{\bf{k}}}\right|\hat{{\rm{S}}}\left|{i}_{1}+{i}_{2},0\right\rangle \right|}^{2}={\left|\left\langle f,{1}_{{\bf{k}}}\right|\hat{{\rm{S}}}\left|{i}_{1},0\right\rangle +\left\langle f,{1}_{{\bf{k}}}\right|\hat{{\rm{S}}}\left|{i}_{2},0\right\rangle \right|}^{2}={\left|{M}_{1}\right|}^{2}+{\left|{M}_{2}\right|}^{2}+2{\mathrm{Re}}\left[{M}_{1}^{* }{M}_{2}\right]$. The last term, $2{\mathrm{Re}}\left[{M}_{1}^{* }{M}_{2}\right]$, is referred to the interference component, which is nonzero only if (1) both transitions $\left|{i}_{1}\right\rangle \to \left|f\right\rangle$ and $\left|{i}_{2}\right\rangle \to \left|f\right\rangle$ emit the same photonic mode ${\bf{k}}$, and (2) ${M}_{1}$, ${M}_{2}$ are not constrained by components that will eliminate each other, such as the Dirac delta (or Kronecker delta). Following the convention in ref. ^2^, for the process solely considering terms ${\left|{M}_{1}\right|}^{2}+{\left|{M}_{2}\right|}^{2}$ (incoherent summation of individual transition probabilities of *|i*_1_〉 and *|i*_2_〉), we refer it as incoherent emission. In contrast, the process considering the full terms is referred as coherent emission, in which the nonzero interference component can significantly affect the final outcome.

In QED, we conventionally consider the cross section $\sigma$, which is proportional to the transition probability, as the observable quantifying the scattering probability of the overall process. Here, we follow the conventions used by Peskin and Schroeder^101^ for the metric tensor, which has diagonal elements $\left\{1,-1,-1,-1\right\}$, and the gamma matrices (shown in Supplementary Section 1). From the Dirac equation, the solution of a single free electron in a definite momentum eigenstate is given by a Dirac planewave ${u}^{s}\left(p\right){e}^{-\frac{i}{\hslash }{p}^{\mu }{x}_{\mu }}$, where ${p}^{\mu }$ is the four-momenta, ${x}_{\mu }$ the position four-vector in spacetime, $\hslash$ the reduced Planck constant, ${u}^{s}\left(p\right)={\left[\sqrt{{p}^{\mu }{\sigma }_{\mu }}{\xi }^{s},\sqrt{{p}^{\mu }{\bar{\sigma }}_{\mu }}{\xi }^{s}\right]}^{{\rm{T}}}/\sqrt{2{p}^{0}}$ the Dirac u-type spinor, ${\sigma }^{\mu }=\left\{1,{\sigma }_{x},{\sigma }_{y},{\sigma }_{z}\right\}$, ${\bar{\sigma }}^{\mu }=\left\{1,{-\sigma }_{x},{-\sigma }_{y},{-\sigma }_{z}\right\}$, ${\sigma }_{x,y,z}$ the $2\times 2$ Pauli matrices, and ${\xi }^{s}$ the spinor where ${\xi }^{\uparrow }={\left[\mathrm{1,0}\right]}^{{\rm{T}}}$ represents spin-up and ${\xi }^{\downarrow }={\left[\mathrm{0,1}\right]}^{{\rm{T}}}$ spin-down. The repeated index convention ${p}^{\mu }{x}_{\mu }={p}^{0}{x}_{0}-{\bf{p}}\cdot {\bf{x}}$ are used throughout the paper, where the bold variables denote the three-vector component of the corresponding four-vectors. The relativistic energy-momentum dispersion relation of the electron is given as ${E}_{p}^{2}={\left({p}^{0}\right)}^{2}{c}^{2}={\left|{\bf{p}}\right|}^{2}{c}^{2}+{m}_{{\rm{e}}}^{2}{c}^{4}$, where ${E}_{p}$ is the electron energy, $c$ is the speed of light in free space and ${m}_{{\rm{e}}}$ is the electron mass. For a photon with four-momenta ${\hslash}k$, its energy ${\hslash}{\omega}_{k}={\hslash}k^0{c}$, where ${\omega }_{k}$ is the angular frequency. The three-momenta, ${\bf{p}}=\{{p}_{x},{p}_{y},{p}_{z}\}$, can be written in spherical coordinates as ${\bf{p}}=\left|{\bf{p}}\right|\left\{\sin \theta \cos \phi ,\sin \theta \sin \phi ,\cos \theta \right\}$, where $\theta$ is the polar angle (incident angle) and $\phi$ is the azimuthal angle. We obtain the expression for bremsstrahlung process involving a single shaped electron wavefunction comprising ${N}_{{\rm{s}}}$ electron momentum states $\mathop{\sum }\nolimits_{m=1}^{{N}_{{\rm{s}}}}{c}_{m}{u}^{{s}_{m}}\left({p}_{m}\right){e}^{-\frac{i}{\hslash }{{p}_{m}}^{\mu }{x}_{\mu }}$ (indices $m$ are positive integers, ${c}_{m}$ is normalized complex coefficient) and arbitrary scatterers comprising ${N}_{{\rm{a}}}$ atomic static potentials $\mathop{\sum }\nolimits_{n=1}^{{N}_{{\rm{a}}}}{A}_{n}\left({\bf{k}}\right)$ (indices *n* are positive integers, **k** is arbitrary wavevector) as

1 $$\begin{array}{l}\frac{{\mathrm{d}}\sigma }{{\mathrm{d}}{\omega}_{{k}^{\prime} }{\mathrm{d}}{\Omega}_{{k}^{\prime}}}=\sum_{{r}^{\prime},{s}^{\prime}}\int {\mathrm{d}}{\Omega}_{{p}^{\prime}}{\delta }_{{E}_{p}-{\hslash}{\omega}_{{k}^{\prime} }-{E}_{{p}^{\prime}}}\frac{{\omega}_{{k}^{\prime}}\left|{\bf{p}}^{\prime} \right|}{8{\varepsilon }_{0}{\left\{2\pi \right\}}^{5}{\hslash}^{3}{c}^{5}\left|{\bf{p}}\right|}\\\qquad\qquad\times {\left|\mathop{\sum }\limits_{n=1}^{{N}_{\mathrm{a}}}\mathop{\sum }\limits_{m=1}^{{N}_{\mathrm{s}}}\left[{A}_{n}\left(\frac{{\bf{p}}_{m}}{\hslash}-{\bf{k}}^{\prime}-\frac{{\bf{p}}^{\prime}}{\hslash}\right)\right]\left[{{c}_{m}{\mathscr{M}}}_{{k}^{\prime}{p}^{\prime} {p}_{m}}^{{r}^{\prime}{s}^{\prime}{s}_{m}}\right]\right|}^{2}\end{array}$$

where primed variables are associated with outgoing particles, ${\Omega }_{{k\text{'}}}$, ${\Omega }_{{p}^{{\prime} }}$ are solid angles of outgoing photon and electron momenta respectively, and ${\delta }_{{E}_{p}-{\hslash} {\omega }_{{k\text{'}}}-{E}_{{p}^{{\prime} }}}$ is the unitless Kronecker delta representing the energy conservation, which constrains the energy of each input electron momentum state to be identical, i.e., ${p}_{m}^{0}c={E}_{p}$ for all $m$. The differential cross section is averaged over the outgoing photon polarization ${r\text{'}}$ and outgoing electron spin ${s\text{'}}$. We obtain the scattering amplitude ${{\mathscr{M}}}_{{k\text{'}}\,{p\text{'}}\,{p}_{m}}^{{r\text{'}}\,{s\text{'}}\,{s}_{m}}$, corresponding to individual transition process from initial state $\left|{p}_{m},{s}_{m};0\right\rangle$ to final state $\left|{p}^{{\prime} },{s}^{{\prime} };{k}^{{\prime} },{r}^{{\prime} }\right\rangle$, as

2 $$\begin{array}{l}{{\mathscr{M}}}_{k^{\prime} \,p^{\prime} \,{p}_{m}}^{r^{\prime} \,s^{\prime} \,{s}_{m}}=-i{q}_{{\rm{e}}}^{2}{\left\{\sqrt{2{{p}^{{\prime} }}^{0}}{u}^{{s}^{{\prime} }}\left({p}^{{\prime} }\right)\right\}}^{\dagger }\left\{{\gamma }^{0}\left({\gamma }^{\sigma }{{\epsilon }_{\sigma }^{{r}^{{\prime} }}}^{* }\right)\frac{{\gamma }^{\nu }{\left(p^{\prime} -{{\hslash }}k^{\prime} \right)}_{\nu }+{m}_{{\rm{e}}}cI}{2{\left(p^{\prime} \right)}^{\mu }{\left({{\hslash }}k^{\prime} \right)}_{\mu }}{\gamma }^{0}\right.\\\qquad\qquad\quad\left.+\frac{{\gamma }^{\nu }{\left[{p}_{m}-{{\hslash }}k^{\prime} \right]}_{\nu }+{m}_{{\rm{e}}}{cI}}{-2{\left({p}_{m}\right)}^{\mu }{\left({{\hslash }}k^{\prime} \right)}_{\mu }}\left({\gamma }^{\sigma }{{\epsilon }_{\sigma }^{{r}^{{\prime} }}}^{* }\right)\right\}\left\{\sqrt{2{p}^{0}}{u}^{{s}_{m}}\left({p}_{m}\right)\right\}\end{array}$$

where ${q}_{{\rm{e}}}$ is the elementary charge, ${\epsilon }^{{r}^{{\prime} }}$ the photon polarization, ${\gamma }^{\nu }$ the gamma matrices and $I$ the $4\times 4$ identity matrix. In the special case of a single scattering potential, our derivation reduces to the known differential cross section ${\rm{d}}\sigma$ for the case in ref. ^2^. Details regarding the derivation of Eqs. (1) and (2) from foundational QED are given in Supplementary Section 1. In the studies presented here, the input electron is always taken as spin-up, i.e., ${s}_{m}= \uparrow$ for all $m$, although our conclusions apply to all initial spin states as we sum the differential cross section over the outgoing photon polarization ${r\text{'}}$ and outgoing electron spin ${s\text{'}}$. As mentioned, due to the Kronecker delta in Eq. (1), $\left|{{\bf{p}}}_{m}\right|$ is fixed for every $m$. The atomic potential contains all momentum components, and thus ensures that momentum is always conserved for the entire process (i.e., compensating the term $\frac{{\bf{p}}_{m}}{\hslash}- {\mathbf{k}}^{\prime}- \frac{{\mathbf{p}}^{\prime}}{\hslash}$ in Eq. (1)), making the bremsstrahlung emission physically possible.

The static potential ${A}_{n}\left({\bf{k}}\right)$ (in Eq. (1)) of the $n$^th^ atom in the scattering material is modeled using a Yukawa potential. The total static potential is thus simply a sum of the static potentials corresponding to each atom. Details of this model are given in Supplementary Section 2. For the shaped single electron wavefunction, we consider a superposition of ${N}_{{\rm{s}}}$ Dirac planewaves, each of which represents an individual electron momentum state with four-momenta ${p}_{m}$:

3 $${\Psi }_{p}\left(x\right)=\mathop{\sum }\limits_{m{\boldsymbol{=}}1}^{{N}_{{\rm{s}}}}{c}_{m}\,{u}^{\uparrow}\left({p}_{m}\right){e}^{-\frac{i}{\hslash}{p}_{m}^{\mu }{x}_{\mu }}$$

where ${c}_{m}$ is the complex coefficient satisfying the normalization condition $\mathop{\sum }\nolimits_{m=1}^{{N}_{{\rm{s}}}}{\left|{c}_{m}\right|}^{2}=1$. The shape of the electron wavefunction, and the corresponding electron spatial probability distribution, can be controlled by varying ${c}_{m}=\left|{c}_{m}\right|{e}^{i{\psi }_{m}}$ via amplitude $\left|{c}_{m}\right|$ and/or phase ${\psi }_{m}={\rm{Arg}}\left({c}_{m}\right)$. Until now, studies on free electron waveshaping enhanced bremsstrahlung have considered only superposition of two momentum states (i.e., ${N}_{{\rm{s}}}=2$)^2^. Here, we increase ${N}_{{\rm{s}}}$ up to hundreds, which provides us with far more degrees of freedom to optimize and shape the single electron wavefunction. Similar to the shaping of optical light^102–106^, one can use Eq. (3) to mathematically construct various kinds of electron beams, including Bessel beams of order $l$ (${c}_{m}={e}^{{il}{\phi }_{m}}$, unnormalized), Hermite Gaussian beams of modes $l$, $q$ (${c}_{m}={(i{p}_{m,x})}^{l}{(i{p}_{m,y})}^{q}$, unnormalized), Laguerre Gaussian beams of modes $l$, $q$ (${c}_{m}={({p}_{m,x}+i{p}_{m,y})}^{l}{({p}_{m,x}-i{p}_{m,y})}^{l+q}$, unnormalized), accelerating beams of order $\alpha$ (${c}_{m}={e}^{i\alpha \theta }$, unnormalized), caustic of mode $l$, $q$ (${c}_{m}={e}^{i\left(l\phi_{m} -q\sin 2\phi_{m} \right)}$^103^$,$ unnormalized), etc. This technique of custom-shaping the electron wavefunction can also be used to achieve probability distributions that overlap with the atomic centers of a crystalline structure, by choosing electron momentum states with transverse wavevectors (momenta) as integer multiples of reciprocal lattice vectors of the corresponding crystal—which for the purposes of simplicity are 2D in the examples presented in this paper. In the discussion, we explain how our findings are readily generalizable to 3D crystals.

**Fig. 1:**
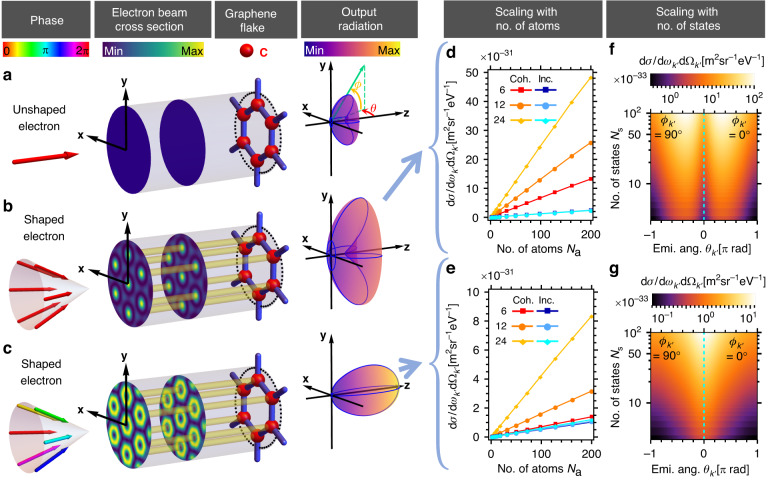
Enhancing both directionality and intensity of bremsstrahlung emission via shaping of electron wavefunction. **a**–**c** illustrate the bremsstrahlung process involving 20 keV initial electron wavefunctions ((**a**) is unshaped, (**b**, **c**) are shaped where (**b**) is converging to a Bessel beam of order 0 and (**c**) is converging to a Bessel beam of order 1, respectively, with increasing number of momentum components ${N}_{{\rm{s}}}$) scattering off a graphene flake (oriented in *xy*-plane), and emitting X-ray photons in multiple directions. Radiation patterns in (**b**, **c**) are distinctly different from (**a**) in terms of both magnitude and directionality, showing the significant effect of quantum interference resulting from electron waveshaping. Compared to (**a**), (**b**) generates radiation of higher intensity while (**c**) generates both stronger and directional radiation (on-axis). (**d**, **e**) show the linear scaling relation between the bremsstrahlung emission differential cross section ${\rm{d}}\sigma /({\rm{d}}{\omega }_{{k}^{{\prime} }}{\rm{d}}{\Omega }_{{k}^{{\prime} }})$ (15 keV photon, at emission angles ${\theta }_{{k}^{{\prime} }}$ = −0.3 [π rad], ${\phi }_{{k}^{{\prime} }}$ = 0.5 [π rad] and ${\theta }_{{k}^{{\prime} }}$ = 0 [π rad], ${\phi }_{{k}^{{\prime} }}$ = 0 [π rad], respectively) and the number of atoms ${N}_{{\rm{a}}}$ for two different shaped electron wavefunctions as mentioned in (**b**, **c**), respectively, for the cases of 6, 12 and 24 momentum states. Similarly, (**f**, **g**) show the scaling relation between the single atom bremsstrahlung differential cross section and the number of electron momentum states ${N}_{{\rm{s}}}$, as mentioned in (**b**, **c**), respectively

We first show that, by increasing the number of atoms ${N}_{{\rm{a}}}$ (i.e., the lattice unit cells covered by the transverse electron beam), we can substantially enhance the differential cross section of bremsstrahlung radiation. We consider a discrete version of electron Bessel beam with wavefunction

4 $${\Psi }_{p}\left(x\right)=\frac{1}{\sqrt{{N}_{s}}}\mathop{\sum }\limits_{m=1}^{{N}_{{\rm{s}}}}{e}^{{il}{\phi }_{m}}{u}^{\uparrow}\left({p}_{m}\left({\theta }_{i},{\phi }_{m}=\frac{m-1}{{N}_{s}}2\pi \right)\right){e}^{-\frac{i}{{{\hslash }}}{p}_{m}^{\mu }{x}_{\mu }}$$

where ${\theta }_{i}$ is the incident angle fixed for all electron momentum states, ${\phi }_{m}$ is the azimuthal angle of the $m$^th^ momentum state, and $l$ is the Bessel order. If ${N}_{{\rm{s}}}$ tends to infinity, the summation becomes continuous integration of the azimuthal angle ${\phi }_{m}\to {\phi }_{i}$, and a typical Bessel beam of order $l$ is obtained (unnormalized). Figure 1b shows a free electron discrete Bessel beam of order 0 that generates bremsstrahlung with an angular profile for which the optimum angle (peak intensity) is not aligned along the electron main propagation direction, i.e., the *z*-axis, which we refer as *off-axis emission*. Conversely, Fig. 1c shows a discrete Bessel beam of order 1 that generates bremsstrahlung with an angular profile for which the photon emission intensity peaks in the direction of the electron wavefunction propagation (referred as *on-axis emission*). As mentioned before, the electron spatial periodic probability distribution matches exactly with the carbon atoms in the graphene lattice as shown in Fig. 1b, c. The differential cross section of the resulting radiation scales linearly with the number of atoms ${N}_{{\rm{a}}}$ as shown in Fig. 1d, e. For a certain photon energy regime (i.e., photon energy <15 keV for a 20 keV electron), one can incoherently sum over the emission rates of bremsstrahlung processes happening at each individual atom to obtain the total differential cross section, and thus obtain the linear ${N}_{{\rm{a}}}$ scaling. We provide a mathematical explanation in Supplementary Section 3 and show that the total differential cross section is obtained with a good approximation, as supported by numerical simulation.

Next, we show the bremsstrahlung differential cross section scales linearly with the number of electron momentum states ${N}_{{\rm{s}}}$ comprising the shaped electron wavefunction. From the examples shown in Fig. 1f, g, the interference between an increasing number of momentum components ${N}_{{\rm{s}}}$ results in a periodic array of approximate Bessel beams, with an increased electron transverse probability distribution profile at each atomic site, leading to the ${N}_{{\rm{s}}}$ linear scaling phenomenon, which saturates when the wavefunction profile converges to a Bessel beam. Unlike the scaling with the number of atoms, this scaling arises due to coherent interference, as can be seen in the comparisons in Fig. 1d, e. With both the ${N}_{{\rm{a}}}$ and ${N}_{{\rm{s}}}$ linear scaling properties, one can generate bremsstrahlung with greatly enhanced intensity in the hard X-ray regime, offering exciting opportunities for novel types of bright X-ray sources.

**Fig. 2:**
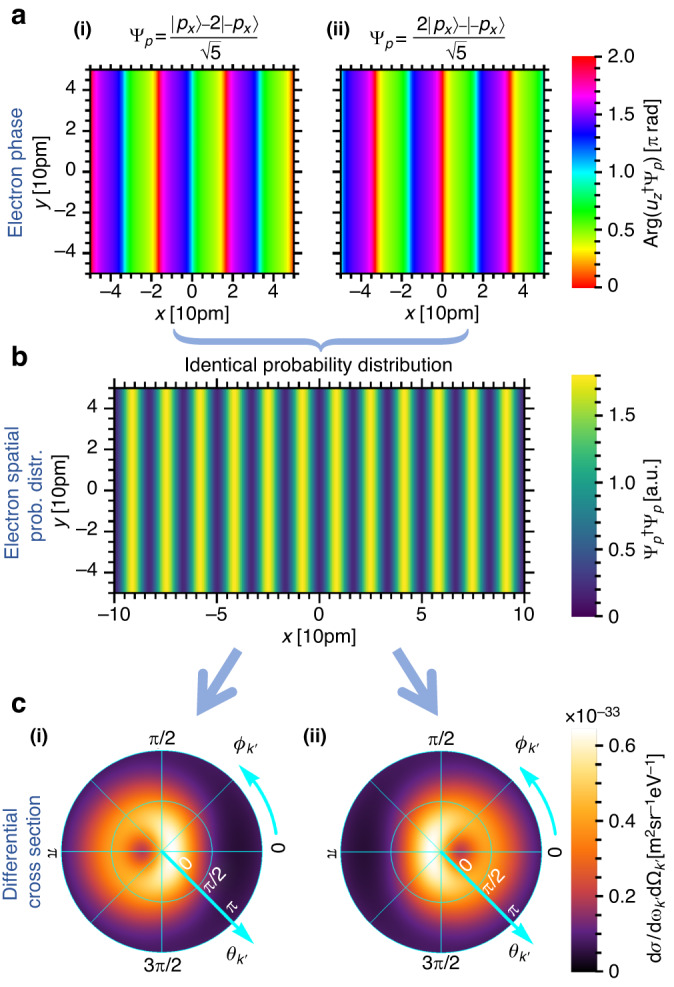
Bremsstrahlung enhancement beyond the free electron spatial probability distribution. We show that non-paraxial electron wavefunctions with identical spatial probability distribution, but different phases, can emit distinctly different radiation patterns. We consider 20 keV shaped electron wavefunctions ${\Psi }_{p}$ with two momentum components (each with an incident angle of 15°), having two different superpositions: $\left(\left|{p}_{x}\right\rangle -2\left|-{p}_{x}\right\rangle \right)/\sqrt{5}$ and $\left(2\left|{p}_{x}\right\rangle -\left|-{p}_{x}\right\rangle \right)/\sqrt{5}$. Their phases ${\rm{Arg}}\left({u}_{z}^{\dagger }{\Psi }_{p}\right)$ (at $z$ = 0) are shown in (**a(i)**, **a(ii)**), respectively, where both of them form identical spatial probability distributions as shown in (**b**). Their corresponding bremsstrahlung emission differential cross sections ${\rm{d}}\sigma /\left({\rm{d}}{\omega }_{{k}^{{\prime} }}{\rm{d}}{\Omega }_{{k}^{{\prime} }}\right)$, for a 15 keV photon, are shown in (**c(i)**, **c(ii)**), respectively. These two examples indicate that the shaping of output radiation does not simply depend on the electron transverse spatial probability distribution and substantially deviate from the prediction under the non-recoil and paraxial approximation

We now further investigate the relation between the initial shaped electron transverse wavefunction and the resulting photon differential cross section. Dependence of spontaneous emission on the free-electron wavefunction has been recently investigated by several authors in the context of coherent cathodoluminescence^107–110^. The main consensus is that in the prevalent paraxial and nonrecoil approximations, wherein the electron initial momentum is orders of magnitude larger than the photon momentum, the emission pattern is given by an incoherent integration over the electron spatial probability distribution and the optical local density of states^111,112^. By extension, one may intuitively expect that the enhancements reported in this paper are simply due to the increased spatial overlap between the electron and the atomic potential. However, careful inspection shows that the bremsstrahlung emission profile does not solely depend on the electron spatial probability distribution in the vicinity of the atoms. Rather, one needs to also consider the relative phases between the electron momentum states. To exemplify this effect, in Fig. 2, we consider two shaped electron wavefunctions with different complex amplitudes, but their spatial probability distributions are analytically identical (Fig. 2b). We show that they generate bremsstrahlung with distinctly different emission profiles oriented in opposite angular directions (Fig. 2c). In order to compare the scalar phases of the two electron wavefunctions, we project their wavefunctions ${\Psi }_{p}$ onto an unshaped single-state electron ${u}_{z}$ with identical energy and momentum solely in *z*-direction. The corresponding arguments are calculated and shown in Fig. 2a. This simple example shows that quantum interference effects in QED resulting from shaped electron wavefunctions should be treated carefully when considering non-paraxial electron wavefunctions (incident angles >15°) and substantial electron recoils (photon energy >75% of the electron kinetic energy). Consequently, the emission profile does not solely depend on the electron spatial probability distribution, and one also needs to consider the electron phase.

**Fig. 3:**
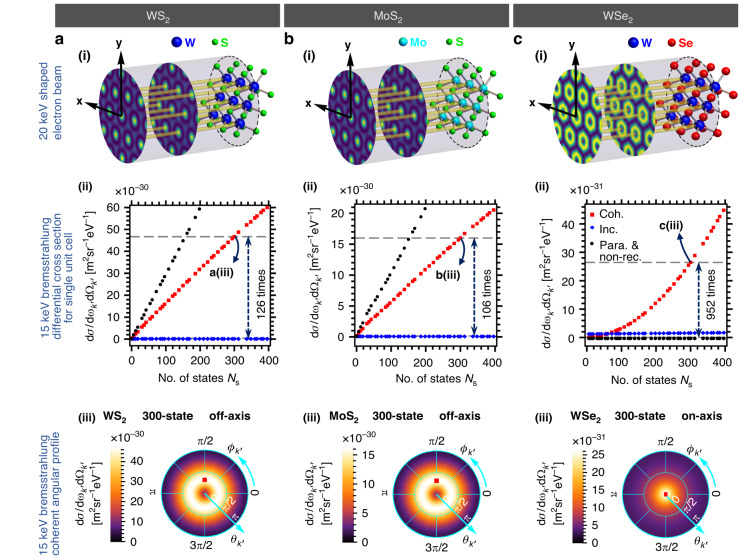
Enhanced bremsstrahlung from heavy-element 2D hexagonal crystal lattices and shaped electron. **a**–**c** illustrate 20 keV, shaped electron wavefunctions with spatial probability distribution localized in the vicinity of the heavier atoms of each crystal lattice unit cell, and their corresponding bremsstrahlung emission profiles at 15 keV. **a(i)**–**c(i)** correspond to WS_2_, MoS_2_, and WSe_2_, respectively, where the electron wavefunction for (**a(i)**, **b(i)**) is an approximate Bessel beam of order 0, and for (**c(i)**) is an approximate Bessel beam of order 1. **a(ii)**–**c(ii)** show the bremsstrahlung differential cross sections for single unit cell against the number of electron momentum states ${N}_{{\rm{s}}}$ for WS_2_, MoS_2_ and WSe_2_ respectively. Optimum photon emission angles are chosen at ${\theta }_{{k}^{{\prime} }}$ = −0.35 [π rad], ${\phi }_{{k}^{{\prime} }}$ = 0.5 [π rad] for **a(ii)** WS_2_ and **b(ii)** MoS_2_ (referred to as *off-axis emission*) and ${\theta }_{{k}^{{\prime} }}$ = 0 [π rad], ${\phi }_{{k}^{{\prime} }}$ = 0 [π rad] for **c(ii)** WSe_2_ (referred to as *on-axis emission*), respectively. For the *off-axis emission* cases **a(ii)** WS_2_ and **b(ii)** MoS_2_, coherent emissions (“Coh.”, red) scale up linearly with the number of states ${N}_{{\rm{s}}}$. Coherent *off-axis emission* is enhanced by more than two orders of magnitude for 300-momentum-state electron, as compared to a single-state (unshaped) electron. The paraxial and non-recoil approximation (“Para. & non-rec.”, black) also predicts linear ${N}_{{\rm{s}}}$ scaling but with a higher intensity. In **c(ii)** WSe_2_, starting from 6-state with a relatively low intensity, coherent *on-axis emission* scales polynomially with the number of states ${N}_{{\rm{s}}}$, showing an enhancement up to three orders of magnitude for 300 electron momentum states. The paraxial and non-recoil approximation fails to produce meaningful results, as it predicts a vanishing emission intensity. In contrast, in all cases, the incoherent emissions (“Inc.”, blue) remain relatively unchanged. **a(iii)**–**c(iii)** show the bremsstrahlung X-ray emission angular profiles (coherent emission) of a 300-momentum-state electron for WS_2_, MoS_2_ and WSe_2_ respectively

Next, we explore bremsstrahlung from more two-dimensional (2D) materials comprising heavier atomic elements. The Yukawa potential generated by an atom is proportional to its atomic number. For bremsstrahlung, the resulting differential cross section is proportional to the square of the atomic number from which the electron wavefunction scatters. Therefore, using 2D materials comprising heavier elements (i.e., tungsten) as scatterer can further enhance the bremsstrahlung output intensity. The natural choice for the candidate is the transition metal dichalcogenide (TMD) monolayers, which have been widely studied and manufactured. The chemical formula of TMD is generally expressed as MX_2_, in which M represents the transition-metal atom (i.e., Mo, W) and X represents chalcogen atom (i.e., S, Se)^113,114^. TMDs are widely applied in optical devices, nanoscale electronic devices, logic gates, memory and spintronics for their unique properties such as direct band gap and strong spin–orbit coupling^113,114^. In our cases, the hexagonal crystalline structure (i.e., lattice constants, lattice vectors) and composition (i.e., atomic number, screening function parameters) of TMDs are our main concerns. For TMDs considered in our model (i.e., WS_2_, MoS_2_ and WSe_2_), although we conventionally refer to them as “monolayer,” each crystal lattice unit cell contains six atoms (two heavier transition-metal atoms and four lighter chalcogen atoms) located at different positions along the longitudinal direction (*z*-axis). We find that it is more convenient and efficient to shape the electron spatial probability distribution profile to the feature patterns periodically overlap with lattice points of the single crystal plane containing solely heavier atoms. This can be achieved by choosing those monoenergetic electron momenta states with transverse wavevectors comprising integer multiples of the reciprocal lattice vectors from the single crystal plane. Theoretically, for WSe_2_ there are around 270 available momentum states (integers of reciprocal lattice wavevectors) for a 20 keV electron with incident angles less than 15°. Details can be found in Supplementary Section 2. In Fig. 3, we demonstrate the scaling properties of the enhanced bremsstrahlung using WS_2_, MoS_2_ and WSe_2_ monolayers. The resulting bremsstrahlung differential cross section for a single unit cell includes the contribution from all 6 atoms in the unit cell. The contribution from one of the two heavier atoms (to which the electron wavefunction is shaped accordingly) becomes dominant at large number of electron momentum states ${N}_{{\rm{s}}}$. In Fig. 3a(ii), b(ii), we plot coherent (red squares) and incoherent (blue diamonds) *off-axis emission* (bremsstrahlung intensity peak is not on the *z*-axis). Both the exact simulation (red squares) and the paraxial & non-recoil approximation (black circles) predicts a ${N}_{{\rm{s}}}$ linear enhancement, but the latter comes with a larger intensity. In Fig. 3c(ii), we plot coherent (red squares) and incoherent (blue diamonds) *on-axis emission* (bremsstrahlung intensity peak is on the *z*-axis). The exact simulation (red squares) shows scaling proportional to ${N}_{{\rm{s}}}$ to the power of roughly 2 while the paraxial and non-recoil approximation (black circles) predicts zero output intensity. Compared to coherent *off-axis emission*, coherent *on-axis emission* is less intense but more directional. The incoherent emissions (blue diamonds) in all cases do not show significant ${N}_{{\rm{s}}}$ dependency and remain stationary as ${N}_{{\rm{s}}}$ increases. This shows that the interference components due to electron waveshaping are essential to the shaping of output photon and significantly depends on ${N}_{{\rm{s}}}$. More details can be found in Supplementary Section 3. The bremsstrahlung differential cross sections for these 2D materials are generally much higher than that for graphene, due to the atoms with larger atomic numbers (tungsten (*Z* = 74), molybdenum (*Z* = 42)) in the 2D materials, compared to carbon (*Z* = 6) in graphene.

**Fig. 4:**
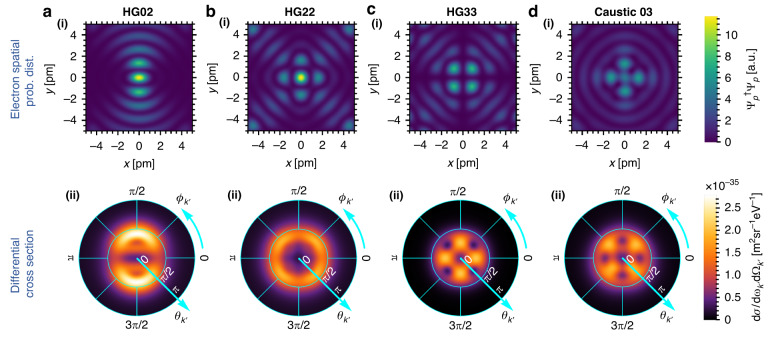
Complex bremsstrahlung emission patterns from different engineered electron wavefunctions. **a(i)**–**d(i)** show the transverse spatial probability distribution (prob. dist.) for a 300 keV electron comprising 20 momentum states (with an incident angle of 0.3 [π rad]), approximating a **a(i)** HG02 beam, **b(i)** HG22 beam, **c(i)** HG33 beam, and **d(i)** Caustic 03 beam^102,103^, respectively. **a(ii)**–**d(ii)** show the corresponding bremsstrahlung differential cross section for 250 keV photon. Precise engineering of electron wavefunctions can, therefore, lead to complex X-ray emission patterns

Finally, we demonstrate how bremsstrahlung can be shaped into complex emission patterns using electron wavefunction shaping beyond the Bessel beam scheme presented above. In Fig. 4, we shape the 300 keV electron wavefunctions using 20 momentum states to form approximate Hermite Gaussian beams of different modes. The shaped bremsstrahlung has angular emission profile that inherits the symmetries of the shaped electron wavefunction (in the *xy*-plane). Note that the examples shown here are highly non-paraxial and contain features down to the picometer scale. Cases of paraxial electron wavefunctions (with incident angles <1°) can be found in Supplementary Section 4, where it is shown that the shaping of bremsstrahlung, to a certain extent, can still be achieved by using a 300 keV electron wavefunction comprising 20 momentum states, having feature sizes around 1 Å.

## Discussion

We have shown that by electron wavefunction shaping, one can greatly enhance the bremsstrahlung differential cross section in the hard X-ray regime by increasing (1) the number of atoms or unit cells in the 2D crystal covered by the electron beam profile, ${N}_{{\rm{a}}}$ and (2) the number of electron momentum states used to approximate the target wavefunction, ${N}_{{\rm{s}}}$. However, one may question the threshold of such enhancements. In principle, the ${N}_{{\rm{a}}}$ scaling is limited by the spatial coherence of the electron beam, i.e., the number of unit cells in the region covered by the electron beam cross section for which the shaped electron wavefunction can still be treated as coherent superposition of multiple planewaves. In short, the larger the electron transverse coherence, the larger the cross section of the periodic electron probability distribution can be maintained, thus covering more atoms and resulting in a higher X-ray intensity. The scaling of X-ray intensity with the number of momentum states ${N}_{{\rm{s}}}$ is limited only by the practical capabilities of electron wavefunction shaping technologies, which has been rapidly advancing in recent years. A 2×2 programmable phase plate using cylindrical electrodes has been reported in 2018^80^ which can generate 300 keV 4-state electron wavefunctions. A very recent work^81^ has demonstrated a 48-element programmable phase plate that can shape electron wavepackets into complex patterns, allowing us (as we show in Supplementary Section 6) to already achieve as much as 18 times intensity enhancement of bremsstrahlung in a proposed experimental demonstration. Research on shaping the electron in 3D to generate a lattice hot-spots has been reported^76^, but the resolution is of tens of nanometers, which is larger than the general crystal lattice constant of few angstroms. New electron waveshaping techniques, such as using short intense laser pulses^66^, surface plasmon polaritons^67^, etc. to modulate electron beam are being developed, and could lead to more versatile ways of spatially shaping the electron wavefunction.

The *off-axis emission* can be understood and approximated employing the paraxial approximation, i.e., treating the scattering elements of each electron momentum states as 1-state (unshaped) scattering element. Under paraxial scheme, one can intuitively consider the bremsstrahlung emission profile is simply proportional to the shaped electron spatial distribution probabilities at each atom. However, the paraxial approximation fails to predict the outcome of *on-axis emission*, for which the bremsstrahlung emission profile is predicted to be extremely low due to the vanishing electron spatial distribution probabilities at each of the atoms. In Fig. 3c, the coherent *on-axis emission* intensity goes further beyond ${N}_{{\rm{s}}}$ linear scaling, where the fitting shows a polynomial scaling in ${N}_{{\rm{s}}}$ to the power of approximately 1.91, starting from 6 states to beyond 400 states, although it does not seem possible to obtain simple analytical approximation to our best knowledge.

For *off-axis emission* examples shown in Fig. 3a(i), b(i), the ratio of bremsstrahlung differential cross sections between 300-state and 1-state electron exceeds 100, which is much lower than the predication (300) following ${N}_{{\rm{s}}}$ scaling. This is simply because for the 1-state scenario, the electron wavefunction is unshaped and its spatial probability distribution is uniform across every atom in the crystal unit cell. Therefore, apart from the heavier atoms, atoms at other layers are contributing significantly to the total bremsstrahlung differential cross section. As the number of electron momentum states increases, contributions from atoms at other layers within the same unit cell become less significant as electron spatial probability distribution vanishes at those atoms’ positions and focused in the vicinity of heavier atoms.

The control over the angular distribution profile of the bremsstrahlung emission via electron waveshaping as shown in Fig. 4 is achieved using electron beam with picometer feature sizes, which is rather hard to achieve with current technology. However, even for more realistic conditions as shown in Fig. S4 (Supplementary Section 4), one can still observe the symmetries between the emission angular distribution profile and the electron transverse spatial probability distribution profile. To the best of our knowledge, there does not exist a simple equation (derived from Eq. (1)) to mathematically describe this relation. Although this limits our way of fully understanding the output emission angular profile, we still have the freedom to shape the electron wavefunctions, which we can still predict the emission outcome to a certain extent.

In this paper, we focused on studying bremsstrahlung from single-layer 2D materials, as opposed to bulk materials. Nevertheless, we emphasize that the concept of shaping bremsstrahlung by shaping the electron wavefunction, as well as the QED framework that we present in this paper, can be generally applied to all configurations of crystalline materials, including bulk crystalline materials. One advantage of using single-layer 2D materials is that electron scattering due to other processes (e.g., Compton scattering), resulting in undesired modification of the electron wavefunction before the desired bremsstrahlung process occurs, can be minimized at any X-ray photon energy. This could lead to lower background noise in the output X-ray spectrum. However, a strong motivation to consider multi-layer 2D materials and bulk materials is to scale up the bremsstrahlung intensity even further, by increasing the chances of bremsstrahlung scattering. For a 3D material case, just as in the 2D material case, we should shape the electron wavefunction such that the shaped electron periodicity matches that of the material’s lattice points. This could be more challenging to achieve with 3D lattices compared to 2D lattices since matching in all three dimensions (as opposed to just two dimensions) of space is needed. Recall that all the electron momentum states should ideally have the same energy, and their choices of transverse momenta are fixed by the reciprocal lattice vectors of the transverse crystal configuration. We thus lose the degree of freedom in controlling the electron longitudinal momentum and the longitudinal spatial probability distribution of the electron. The use of multilayer 2D heterostructures with controllable interlayer spacings, e.g., twisted bilayers and multi-layers, could provide a solution to this problem, since the different layers can be inverse designed to match the electron spatial longitudinal profile. In Supplementary Section 5, we present an example showing how our concept of enhancing bremsstrahlung by shaping the electron wavefunction applies readily to the case of multi-layer and bulk materials: in this case, the scaling up of bremsstrahlung with the number of layers was achieved by designing an electron wavefunction which is invariant along the depth dimension of the 3D material.

We note that the apparatus and parameters needed for experimental demonstrations are well within the state-of-the-art. In Supplementary Section 6, we propose specific experimental plans using currently available apparatus, to show that it is already possible to demonstrate up to 18 times enhancement in bremsstrahlung intensity, and observable changes in the bremsstrahlung angular pattern from electron waveshaping. These findings in turn motivate further research and development in the technologies involved—including electron waveshaping elements like multi-element phase plates, as well as nanopositioning stages.

In conclusion, our work shows that tunable bremsstrahlung in the hard X-ray regime can be enhanced by over three orders of magnitude via quantum interference enabled by electron waveshaping. Specifically, we shape the electron spatial probability distribution profile to exactly match the periodic crystalline structure of the 2D atomic centers. This approach may appear intuitive based on the logic of maximizing the probability of the electron traveling near the atoms, but our studies show that the phenomenon goes beyond this trivial explanation: In fact, we prove that both the electron spatial probability distribution profile and electron wavefront are essential to the final output of shaped bremsstrahlung process, thus highlighting the limitations of the paraxial approximation and non-recoil assumption. The resulting bremsstrahlung emission can scale up almost quadratically with the number of electron momentum states and linearly with the number of atomic centers involved in the emission process. We showcase our ability to manipulate the angular distribution profile of bremsstrahlung, resulting in better directionality or any other intended shape. Our results pave the way to bespoke, enhanced sources of bremsstrahlung, as well as multi-dimensional control of scatterers in general QED free electron emission processes.

### Supplementary information

Supplementary Information: Free-electron crystals for enhanced X-ray radiation
