# Supplementary material for: Free-electron crystals for enhanced X-ray radiation
Source: Light Sci Appl. 2024 Jan 24;13:29. doi: 10.1038/s41377-023-01363-4 (PMC10808554; doi:10.1038/s41377-023-01363-4)
Supplement: Supplementary file 1 — Supplementary Information: Free-electron crystals for enhanced X-ray radiation [file 41377_2023_1363_MOESM1_ESM.pdf]

Supplementary Information:
Free-electron crystals for enhanced X-ray radiation

Lee Wei Wesley Wong[1], Xihang Shi[2], Aviv Karnieli[3,4], Jeremy Lim[5], Suraj Kumar[1], Sergio Carbajo[6,7,8], Ido Kaminer[2] & Liang Jie Wong[1,*]

[1]*School of Electrical and Electronic Engineering, Nanyang Technological University, 50 Nanyang Avenue, Singapore 639798, Singapore*

[2]*Solid State Institute and Faculty of Electrical and Computer Engineering, Technion – Israel Institute of Technology, Haifa 3200003, Israel*

[3]*School of Electrical Engineering, Fleischman Faculty of Engineering, Tel Aviv University, Tel Aviv 69978, Israel*

[4]*Department of Applied Physics, Stanford University, Stanford, CA 94305, USA*

[5]*Science, Mathematics and Technology, Singapore University of Technology and Design, 8 Somapah Road, Singapore 487372, Singapore*

[6]*Electrical and Computer Engineering Department, UCLA, 420 Westwood, Los Angeles, CA 90095, USA*

[7]*Physics and Astronomy Department, UCLA, 475 Portola Plaza, Los Angeles, CA 90095, USA*

[8]*SLAC National Accelerator Laboratory, Stanford University, 2575 Sand Hill Rd, Menlo Park, CA 94025, USA*

[*]*Email: liangjie.wong@ntu.edu.sg*

Table of Contents

Section 1 | Atomic bremsstrahlung with shaped electron wavefunctions 2

Section 2 | Yukawa potential and crystal structure 6

Yukawa potential of crystal lattice 6

Crystalline structure and reciprocal lattice vectors 8

Section 3 | Simplified models based on approximations related to coherence, paraxiality and recoil 11

Coherent and incoherent emission 11

Incoherent summation of number of atoms N_a 12

Paraxial approximation 14

Non-recoil approximation 16

Section 4 | Further examples of tailoring bremsstrahlung via electron waveshaping 17

Section 5 | Enhancement of bremsstrahlung X-ray in 3D bulk crystalline materials 18

Section 6 | Experimental proposals for proof-of-concept demonstrations 19

References 23

Section 1 | Atomic bremsstrahlung with shaped electron wavefunctions

Differential cross section of bremsstrahlung emission

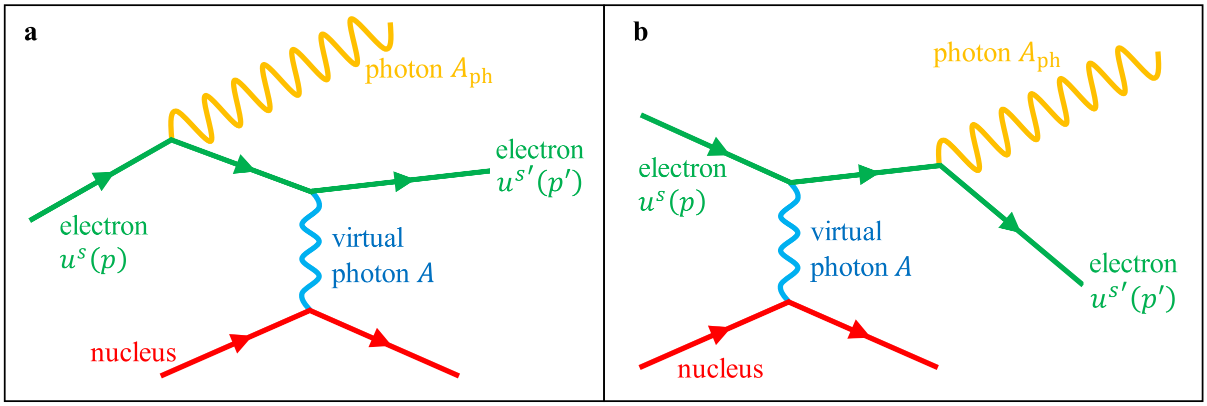

Fig. S1 Feynman diagram for atomic bremsstrahlung.

Bremsstrahlung is a typical second-order QED process, which an input electron $\frac{1}{\sqrt{2p^0}}e^{-\frac{i}{\hbar}p^\mu x_\mu}u^s(p)$ (four-momentum p and spin s) is scattered by potential $A(x)$ (nucleus) into output electron $\frac{1}{\sqrt{2p'^0}}e^{-\frac{i}{\hbar}p'^\mu x_\mu}u^{s'}(p')$ (four-momentum p' and spin s') and emitting photon $A_{\rm ph}^{r'}(k',x)$ (four-momentum $\hbar k'$ and polarization r'). The transition matrix element $M_{k'p'p}^{r's's}$ is given by

$$M_{k'p'p}^{r's's} = \int_{-\infty}^{\infty} \mathrm{d}^4x\, \mathrm{d}^4y \left\{\frac{1}{\sqrt{V}}\frac{1}{\sqrt{2p'^0}}e^{-\frac{i}{\hbar}p'^\mu x_\mu}u^{s'}(p')\right\}$$
$$\times\left\{\underbrace{\left[i\frac{q_{\rm e}}{\hbar}\gamma^\mu A_{\rm ph}^{r'}(k',x)_\mu\right][iS_{\rm F}(x-y)]\left[i\frac{q_{\rm e}}{\hbar}\gamma^\nu A(y)_\nu\right]}_{\text{Correspond to Fig. S1b}}\right. \tag{S1.1}$$
$$\left.+\underbrace{\left[i\frac{q_{\rm e}}{\hbar}\gamma^\mu A(x)_\mu\right][iS_{\rm F}(x-y)]\left[i\frac{q_{\rm e}}{\hbar}\gamma^\nu A_{\rm ph}^{r'}(k',y)_\nu\right]}_{\text{Correspond to Fig. S1a}}\right\}$$
$$\times\left\{\frac{1}{\sqrt{V}}\frac{1}{\sqrt{2p^0}}e^{-\frac{i}{\hbar}p^\mu x_\mu}u^s(p)\right\}\quad,$$

where spacetime integral $\int_{-\infty}^{\infty} \mathrm{d}^4x = \int_{-\infty}^{\infty} \mathrm{d}t \int_{-\infty}^{\infty} \mathrm{d}^3\mathbf{r}$, q_e is the elementary charge and V is the interaction volume. We follow metric tensor and gamma matrix conventions of ref. [1], relevant details are listed below:

Pauli matrices:

$$\sigma^\mu = \{1, \sigma_x, \sigma_y, \sigma_z\}, \qquad \sigma_x = \begin{pmatrix} 0 & 1 \\ 1 & 0 \end{pmatrix}, \qquad \sigma_y = \begin{pmatrix} 0 & -i \\ i & 0 \end{pmatrix}, \qquad \sigma_z = \begin{pmatrix} 1 & 0 \\ 0 & -1 \end{pmatrix}; \tag{S1.2}$$

gamma matrices:

$$\gamma^0 = \begin{pmatrix} 0_{2\times2} & 1_{2\times2} \\ 1_{2\times2} & 0_{2\times2} \end{pmatrix}, \qquad \gamma^j = \begin{pmatrix} 0_{2\times2} & \sigma^j \\ -\sigma^j & 0_{2\times2} \end{pmatrix}, \qquad j = 1, 2, 3; \tag{S1.3}$$

electron Dirac four-vector:

$$u^s(p) = \begin{pmatrix} \sqrt{p^\mu \sigma_\mu} \xi^s \\ \sqrt{p^\mu \bar{\sigma}_\mu} \xi^s \end{pmatrix} = \frac{\begin{pmatrix} \{\{p^0 + m_\mathrm{e} c\} I - \mathbf{p} \cdot \boldsymbol{\sigma}\} \xi^s \\ \{\{p^0 + m_\mathrm{e} c\} I + \mathbf{p} \cdot \boldsymbol{\sigma}\} \xi^s \end{pmatrix}}{\sqrt{2\{p^0 + m_\mathrm{e} c\}}}, \qquad \bar{u}^{s'}(p') = \{u^{s'}(p')\}^\dagger \gamma^0; \tag{S1.4}$$

photon four-vector:

$$A_\mathrm{ph}^{r'}(k', x) = \sqrt{\frac{\hbar}{2\varepsilon_0 \omega_{k'} V}} \epsilon^{r'} e^{-ik'^\mu x_\mu}, \qquad r' = 1,2; \tag{S1.5}$$

photon polarization four-vector:

$$\epsilon^1 = \begin{pmatrix} 0 \\ \cos\theta_{k'} \cos\phi_{k'} \\ \cos\theta_{k'} \sin\phi_{k'} \\ -\sin\theta_{k'} \end{pmatrix}, \qquad \epsilon^2 = \begin{pmatrix} 0 \\ \sin\phi_{k'} \\ -\cos\phi_{k'} \\ 0 \end{pmatrix},$$
$$k^\mu \epsilon_\mu^{r'} = 0, \qquad \epsilon^{r_1'{}^\mu} \epsilon_\mu^{r_2'} = -\delta_{r_1', r_2'}, \qquad r_1', r_2' = 1,2; \tag{S1.6}$$

Dirac propagators:

$$\mathrm{S_F}(x) = \int_{-\infty}^{\infty} \frac{\mathrm{d}^4 q}{\{2\pi\hbar\}^4} \frac{e^{-\frac{i}{\hbar} q^\mu x_\mu}}{\frac{1}{\hbar} \gamma^\nu q_\nu - \frac{m_\mathrm{e} c}{\hbar} I}; \tag{S1.7}$$

where q^μ has the form of momentum four-vector. In our model we consider Yukawa potential as the static field and its potential four-vector has the form $A(x) = \{A_\mathrm{Yu}(\mathbf{x}), 0, 0, 0\}$. Details for Yukawa potential $A_\mathrm{Yu}(\mathbf{x})$ can be found in Supp. Sect. 2.

From equation (S1.1) we obtain

$$M_{k'p'p}^{r's's} = \sqrt{\frac{1}{V^3}\frac{\hbar c^2}{8\varepsilon_0\omega_{k'}E_{p'}E_p}}2\pi\delta\big[E_p - \hbar\omega_{k'} - E_{p'}\big]A_{\mathrm{Yu}}(\boldsymbol{\kappa})\mathcal{M}_{k'p'p}^{r's's}\,, \tag{S1.8}$$

where $A_{\mathrm{Yu}}(\mathbf{k})$ is the Fourier transform of $A_{\mathrm{Yu}}(\mathbf{x})$ and $\boldsymbol{\kappa} = \frac{\mathbf{p}}{\hbar} - \mathbf{k}' - \frac{\mathbf{p}'}{\hbar}$. The Dirac delta distribution $\delta\big[E_p - \hbar\omega_{k'} - E_{p'}\big]$ represents the energy conservation $E_p = E_{p'} + \hbar\omega_{k'}$, and

$$\mathcal{M}_{k'p'p}^{r's's} = -\mathrm{i}q_{\mathrm{e}}^2\{u^{s'}(p')\}^\dagger\left\{\gamma^0\left(\gamma^\mu\epsilon_\mu^{r'*}\right)\frac{\gamma^\nu(p'+\hbar k')_\nu + m_{\mathrm{e}}cI}{2(p')^\mu(\hbar k')_\mu}\gamma^0 + \frac{\gamma^\nu(p-\hbar k')_\nu + m_{\mathrm{e}}cI}{-2p^\mu(\hbar k')_\mu}\left(\gamma^\mu\epsilon_\mu^{r'*}\right)\right\}\{u^s(p)\}\ . \tag{S1.9}$$

Note that equation (S1.9) has the same form as equation (2) in the main text.

The cross section σ of bremsstrahlung involving a single-shaped electron wavefunction comprising N_{s} electron momentum states $\sum_{m=1}^{N_{\mathrm{s}}} c_m u^{s_m}(p_m)e^{-\frac{i}{\hbar}p_m{}^\mu x_\mu}$ (complex coefficients c_m) can be expressed as

(1) the coherent summation over the different transition matrix elements $M_{k'p'p_m}^{r's's_m}$ associated with each input electron momentum state p_m and spin s_m, and
(2) an incoherent summation over all possible final electron momentum states p' and spin s', corresponding to trace out the final free-electron degrees of freedom, as the latter is not measured,

given by

$$\sigma = \frac{V^3}{vT}\int\frac{\mathrm{d}^3\mathbf{p}'}{\{2\pi\hbar\}^3}\frac{\mathrm{d}^3\mathbf{k}'}{\{2\pi\}^3} \times \sum_{r',s'}\left|\sqrt{\frac{1}{V^3}\frac{\hbar c^2}{8\varepsilon_0\omega_{k'}E_{p'}E_p}}2\pi\delta\big[E_p - \hbar\omega_{k'} - E_{p'}\big]\sum_{m=1}^{N_{\mathrm{s}}}A_{\mathrm{Yu}}(\boldsymbol{\kappa}_m)c_m\mathcal{M}_{k'p'p_m}^{r's's_m}\right|^2 \tag{S1.10}$$

where T is the interaction time, v is the speed of input particles and $\boldsymbol{\kappa}_m = \frac{\mathbf{p}_m}{\hbar} - \mathbf{k}' - \frac{\mathbf{p}'}{\hbar}$. Note that the Dirac delta distribution $\delta\big[E_p - \hbar\omega_{k'} - E_{p'}\big]$ only keep those electron

momentum states with identical energy E_p. Therefore, only those electron momentum states can effectively interfere with each other and affect the cross section.

The absolute square of Dirac delta can be recast as

$$\begin{aligned}
\left|2\pi\delta\left[E_p - \hbar\omega_{k'} - E_{p'}\right]\right|^2 &= \lim_{T\to\infty} \int_{-\frac{T}{2}}^{\frac{T}{2}} \frac{\mathrm{d}t}{\hbar} e^{i\left(E_p - \hbar\omega_{k'} - E_{p'}\right)\frac{t}{\hbar}} 2\pi\delta\left[E_p - \hbar\omega_{k'} - E_{p'}\right] \\
&= \lim_{T\to\infty} \frac{T}{\hbar} 2\pi\delta\left[E_p - \hbar\omega_{k'} - E_{p'}\right] \\
&= \lim_{T\to\infty} \frac{T}{\hbar} \frac{2\pi\delta[|\mathbf{p}'| - |\mathbf{p}'|_0\,]}{\left|\left[\frac{\mathrm{d}\left(E_p - \hbar\omega_{k'} - E_{p'}\right)}{\mathrm{d}|\mathbf{p}'|}\right]_{|\mathbf{p}'|=|\mathbf{p}'|_0}\right|} \\
&= \lim_{T\to\infty} \frac{T}{\hbar} 2\pi \frac{\delta[|\mathbf{p}'| - |\mathbf{p}'|_0\,]}{\frac{|\mathbf{p}'|_0 c^2}{E_{p'}{}_0}}
\end{aligned} \tag{S1.11}$$

where $E_{p'} = \sqrt{|\mathbf{p}'|^2 c^2 + (m_\mathrm{e} c^2)^2}$, $E_{p'}{}_0 = E_p - \hbar\omega_{k'} = \sqrt{|\mathbf{p}'|_0{}^2 c^2 + (m_\mathrm{e} c^2)^2}$.

Recasting the integrals $\int \mathrm{d}^3\mathbf{p}' = \int |\mathbf{p}'|^2 \mathrm{d}|\mathbf{p}'| \mathrm{d}\Omega_{p'}$ and $\int \mathrm{d}^3\mathbf{k}' = \frac{1}{c^3}\int (\omega_{k'})^2 \mathrm{d}\omega_{k'} \mathrm{d}\Omega_{k'}$ (solid angles $\Omega_{p'}$, $\Omega_{k'}$ and $\omega_{k'} = |\mathbf{k}'|c$), and speed $v = \frac{|\mathbf{p}|c^2}{E_p}$, we proceed to compute the differential cross section (cross section σ per unit angular frequency $\omega_{k'}$ per unit solid angle $\Omega_{k'}$) as

$$\begin{aligned}
\frac{\mathrm{d}\sigma}{\mathrm{d}\omega_{k'}\mathrm{d}\Omega_{k'}} &= \frac{\omega_{k'}}{8\varepsilon_0 \{2\pi\}^5 \hbar^3 c^5 |\mathbf{p}|} \int \mathrm{d}\Omega_{p'} \\
&\quad \times \int \mathrm{d}|\mathbf{p}'| \sum_{r',s'} \frac{E_{p'}{}_0}{E_{p'}} \frac{|\mathbf{p}'|^2}{|\mathbf{p}'|_0} \delta[|\mathbf{p}'| - |\mathbf{p}'|_0\,] \left| \sum_{m=1}^{N_\mathrm{s}} A_\mathrm{Yu}(\boldsymbol{\kappa}_m) c_m \mathcal{M}_{k'p'p_m}^{r's's_m} \right|^2 ,
\end{aligned} \tag{S1.12}$$

carrying out the integral of Dirac delta we finally obtain

$$\frac{\mathrm{d}\sigma}{\mathrm{d}\omega_{k'}\mathrm{d}\Omega_{k'}}$$

$$= \sum_{r',s'} \int \mathrm{d}\Omega_{p'} \delta_{E_p - \hbar\omega_{k'} - E_{p'}} \frac{\omega_{k'} |\mathbf{p}'|_0}{8\varepsilon_0 \{2\pi\}^5 \hbar^3 c^5 |\mathbf{p}|} \left| \sum_{m=1}^{N_\mathrm{s}} A_\mathrm{Yu}(\boldsymbol{\kappa}_m) \left[c_m \mathcal{M}_{k'p'p_m}^{r's's_m} \right] \right|^2 \qquad \text{(S1.13)}$$

where we purposely insert unitless Kronecker delta $\delta_{E_p - \hbar\omega_{k'} - E_{p'}}$ as a reminder for energy conservation. Note that equation (S1.13) is the same as equation (1) in main text, only the Yukawa potential $A_\mathrm{Yu}(\boldsymbol{\kappa}_m)$ is not explicitly written out in summation form.

Section 2 | Yukawa potential and crystal structure

Yukawa potential of crystal lattice

For an arbitrary crystal lattice containing N_a atoms, the total Yukawa potential $A_\mathrm{Yu}(\mathbf{x})$ in space domain can be expressed as

$$A_\mathrm{Yu}(\mathbf{x}) = \sum_{n=1}^{N_\mathrm{a}} \sum_{j_n} \frac{-Z_n q_\mathrm{e}}{4\pi\varepsilon_0} \frac{C_{j_n} e^{-\frac{\mu_{j_n}}{a_0}|\mathbf{x}-\mathbf{d}_n|}}{|\mathbf{x}-\mathbf{d}_n|}, \qquad \text{(S2.1)}$$

where q_e is the elementary charge, ε_0 is the vacuum permittivity, a_0 is the Bohr radius and $\mathbf{x}$ denotes the position vector. For n^th atom in the crystal lattice, Z_n denotes the atomic number, $\mathbf{d}_n$ is the lattice vector, C_{j_n} and μ_{j_n} are the screening function parameters analytically fitted to Dirac-Hartree-Fock-Slater (DHFS) self-consistent data as given in refs [2,3]. In our study, we consider 2D crystalline materials including graphene, WS_2, $MoSe_2$ and WSe_2. Information regarding crystal structures and lattice parameters used in our calculation are from The Materials Project [4]. Other relevant physical parameters are listed in Table 1.

Table 1. Atomic numbers and screening function parameters.

Element	Z	C_1	C_2	C_3	μ_1	μ_2	μ_3
C	6	0.1537	0.8463		8.0404	1.4913	
S	16	0.5459	−0.5333	0.9874	6.3703	2.5517	1.6753
Se	34	0.4836	0.5164		8.7824	1.6967	
Mo	42	0.2693	0.5763	0.1544	14.044	2.8611	1.0591
W	74	0.1500	0.6871	0.1629	28.630	4.2426	1.2340

In the calculation we need to Fourier transform the Yukawa potential into momentum space. Utilizing the infinite space integral, one can transform the Cartesian coordinate $\mathbf{x} - \mathbf{d}_n$ to drop the $\mathbf{d}_n$ such that for arbitrary wavevector $\mathbf{k}$, the Fourier transform is computed:

$$\int_{-\infty}^{\infty} \mathrm{d}^3\mathbf{x} \frac{e^{-\frac{\mu_{j_n}}{a_0}|\mathbf{x}-\mathbf{d}_n|} e^{i\mathbf{k}\cdot\mathbf{x}}}{|\mathbf{x}-\mathbf{d}_n|} \rightarrow \int_{-\infty}^{\infty} \mathrm{d}^3\mathbf{x} \frac{e^{-\frac{\mu_{j_n}}{a_0}|\mathbf{x}|} e^{i\mathbf{k}\cdot(\mathbf{x}+\mathbf{d}_n)}}{|\mathbf{x}|} = e^{i\mathbf{k}\cdot\mathbf{d}_n} \int_{-\infty}^{\infty} \mathrm{d}^3\mathbf{x} \frac{e^{-\frac{\mu_{j_n}}{a_0}|\mathbf{x}|+i\mathbf{k}\cdot\mathbf{x}}}{|\mathbf{x}|} \quad . \tag{S2.2}$$

Without loss of generality we can always choose the Cartesian coordinate system such that the *z*-axis is parallel to the wavevector $\mathbf{k}$. One can then simplify the integrals by tranforming Eq (S2.2) into spherical coordinate system:

$$e^{i\mathbf{k}\cdot\mathbf{d}_n} \int_{-\infty}^{\infty} \mathrm{d}^3\mathbf{x} \frac{e^{-\frac{\mu_{j_n}}{a_0}|\mathbf{x}|+i\mathbf{k}\cdot\mathbf{x}}}{|\mathbf{x}|} = e^{i\mathbf{k}\cdot\mathbf{d}_n} \int_0^{\infty} \mathrm{d}r \int_0^{\pi} \mathrm{d}\theta \int_0^{2\pi} \mathrm{d}\phi \, r^2 \sin\theta \frac{e^{-\frac{\mu_{j_n}}{a_0}r+i|\mathbf{k}|r\cos\theta}}{r} \quad . \tag{S2.3}$$

The Yukawa potential in Fourier space is given by

$$\begin{aligned} A_{\mathrm{Yu}}(\mathbf{k}) &= \sum_{n=1}^{N_\mathrm{a}} A_n(\mathbf{k}) = \sum_{n=1}^{N_\mathrm{a}} a_n(\mathbf{k}) e^{i\mathbf{k}\cdot\mathbf{d}_n} \quad , \\ a_n(\mathbf{k}) &= \sum_{j_n} \frac{-Z_n q_\mathrm{e}}{\varepsilon_0} \frac{C_{j_n}}{\left(\frac{\mu_{j_n}}{a_0}\right)^2 + |\mathbf{k}|^2} \in \mathbb{R} \quad . \end{aligned} \tag{S2.4}$$

Crystalline structure and reciprocal lattice vectors

For the first example in the main text (Fig. 1) we choose the 2D graphene (oriented in the xy-plane) as our scatterer for bremsstrahlung.

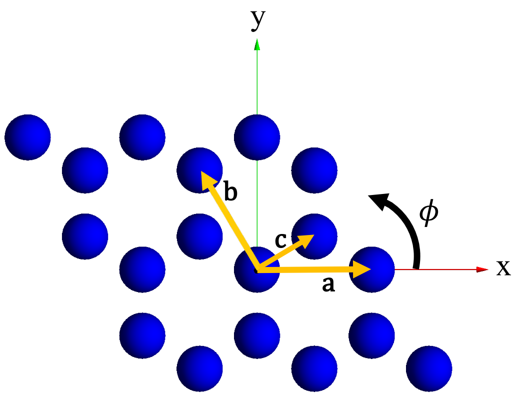

Fig. S2 Illustration of graphene.

In Fig. S2, the graphene (or other 2D crystal lattice with hexagonal structure) is oriented in xy-plane, with lattice basis vectors **a**, **b** and **c** and lattice constant a:

$$|\mathbf{a}| = |\mathbf{b}| = a, \quad |\mathbf{c}| = \frac{\sqrt{3}}{3}a, \quad \measuredangle_{\mathbf{ab}} = 120°, \quad \measuredangle_{\mathbf{ac}} = \measuredangle_{\mathbf{cb}} = 30°,$$
$$\mathbf{a} = a\{1,\ 0,\ 0\}, \quad \mathbf{b} = a\left\{-\frac{1}{2},\ \frac{\sqrt{3}}{2},\ 0\right\}, \quad \mathbf{c} = a\left\{\frac{1}{2},\ \frac{\sqrt{3}}{6},\ 0\right\}, \tag{S2.5}$$

Each unit cell contains 2 carbon atoms, therefore, for n^{th} atom in the crystal lattice, its lattice vector $\mathbf{d}_n$ can always be expressed as $\mathbf{d}_n = n_1\mathbf{a} + n_2\mathbf{b}$ or $\mathbf{d}_n = \mathbf{c} + n_1\mathbf{a} + n_2\mathbf{b}$, where $n_1, n_2 \in \mathbb{Z}$. The former gives reciprocal lattice vectors

$$\mathbf{G}_1 = 2\pi\frac{\mathbf{b}\times\{0,\ 0,\ 1\}}{\mathbf{a}\cdot(\mathbf{b}\times\{0,\ 0,\ 1\})} = \frac{2\pi}{a}\left\{1,\ \frac{\sqrt{3}}{3},\ 0\right\}, \tag{S2.6}$$

$$\mathbf{G}_2 = 2\pi\frac{\{0,\ 0,\ 1\}\times\mathbf{a}}{\mathbf{a}\cdot(\mathbf{b}\times\{0,\ 0,\ 1\})} = \frac{2\pi}{a}\left\{0,\ \frac{2\sqrt{3}}{3},\ 0\right\}, \tag{S2.7}$$

and the latter gives reciprocal lattice vectors

$$\mathbf{G}_1' = 2\pi\frac{(\mathbf{b}+\mathbf{c})\times\{0,\ 0,\ 1\}}{(\mathbf{a}+\mathbf{c})\cdot\big((\mathbf{b}+\mathbf{c})\times\{0,\ 0,\ 1\}\big)} = \frac{2\pi}{a}\left\{\frac{2}{3},\ 0,\ 0\right\}, \tag{S2.8}$$

$$\mathbf{G}_2' = 2\pi\frac{\{0,\ 0,\ 1\}\times(\mathbf{a}+\mathbf{c})}{(\mathbf{a}+\mathbf{c})\cdot\big((\mathbf{b}+\mathbf{c})\times\{0,\ 0,\ 1\}\big)} = \frac{2\pi}{a}\left\{-\frac{1}{6},\ \frac{\sqrt{3}}{2},\ 0\right\}, \tag{S2.9}$$

We can construct shaped electron wavefunctions via selecting those electron momentum states with transverse wavevectors $\mathbf{p}_{\perp,m}/\hbar = m_1\mathbf{G}_1 + m_2\mathbf{G}_2$ (or $\mathbf{p}_{\perp,m}/\hbar = m_1\mathbf{G}_1' + m_2\mathbf{G}_2'$), where $m_1, m_2 \in \mathbb{Z}$. The resulting electron spatial probability distribution possesses the same periodicity as those atoms with lattice vectors $\mathbf{d}_n = n_1\mathbf{a} + n_2\mathbf{b}$ (or $\mathbf{d}_n = \mathbf{c} + n_1\mathbf{a} + n_2\mathbf{b}$). For a "real" 2D crystal, i.e. all atoms lie in same plane such as graphene, there are conditions where one can choose wavevectors $\mathbf{p}_{\perp,m}/\hbar = m_1\mathbf{G}_1 + m_2\mathbf{G}_2 = m_1'\mathbf{G}_1' + m_2'\mathbf{G}_2'$ for some particular integers m_1, m_2, m_1', m_2'. The resulting electron spatial probability distribution possesses the same periodicity as all the atoms, but also note that since some of the choices of reciprocal lattice vectors are omitted, the density of available momentum states also decreases.

For other 2D materials considered in our study, i.e., WS_2 (a = 3.1907Å), $MoSe_2$ (a = 3.3269Å), and WSe_2 (a = 3.3271Å), they have similar hexagonal structure as graphene (a = 2.4680Å), but each atoms in the unit cell (total 6 atoms) is located at different layer along longitudinal direction (z-axis). Therefore, we only choose the reciprocal lattice vectors from one of the layers of heavier atoms (transition-metal) as the basic of selecting the suitable electron momentum states. In Fig. S3, the black dots represent reciprocal lattice vectors of one layer of tungsten atoms (with lattice vectors $m_1\mathbf{G}_1 + m_2\mathbf{G}_2$ for integers m_1, m_2) in the unit cell of WSe_2. The black dots falling in the same circle have the same magnitude. For electron momentum states with fixed energy (20 keV in Fig. S2), we can only choose those with transverse wavevectors denoted by the black dots, the incident angle θ of the electron momentum state can be calculated accordingly.

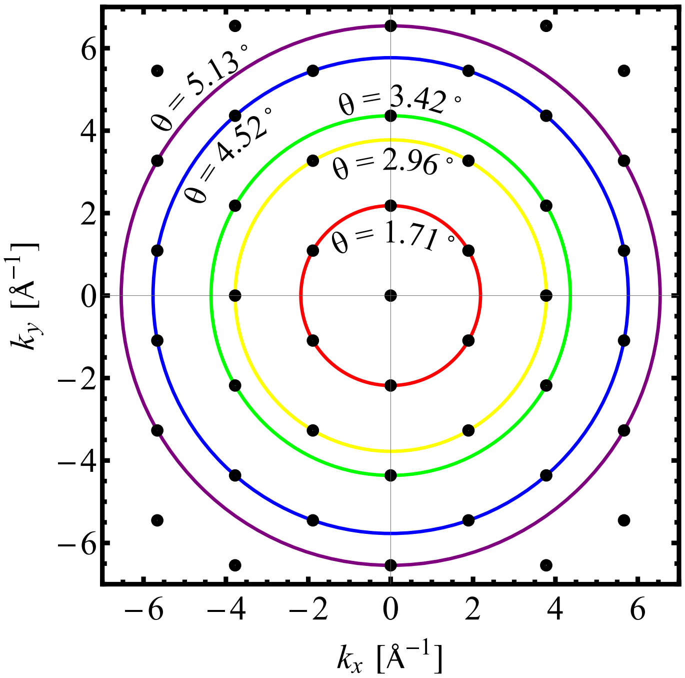

Fig. S3 Reciprocal lattice vectors of one layer of tungsten atoms in the unit cell of WSe_2. The θ denotes the incident angle of a 20 keV electron momentum state with transverse wavevectors falling in the corresponding circles. The first circle (red) has 6 available wavevectors, the second circle (yellow) has another 6 available wavevectors, etc.

Section 3 | Simplified models based on approximations related to coherence, paraxiality and recoil

Coherent and incoherent emission

Considering a Dirac electron wavefunction $\Psi_p(x)$ comprising a discrete superposition of N_s plane waves with different four-momenta p_m, and identical energy E_p and spin s:

$$\Psi_p(x) = \sum_{m=1}^{N_\mathrm{s}} c_m e^{-\frac{i}{\hbar}p_m^\mu x_\mu}\, u^s(p_m)\,, \quad p_m^0 = \frac{E_p}{c} \text{ for all } m, \tag{S3.1}$$

where each discrete electron momentum state is associated with the normalized complex coeffiecient

$c_m = |c_m|e^{i\psi_m}$ (amplitude $|c_m|$ and phase ψ_m). We assume that all the input electron momentum states are spin-up, i.e., $s = \uparrow$ for all m throughout the paper.

The spatial probability distribution function of electron wavefunction $\Psi_\mathrm{i}^\dagger\Psi_\mathrm{i}(\mathbf{x})$ can be written as

$$\Psi_p^\dagger\Psi_p(\mathbf{x}) = \sum_{m=1}^{N_\mathrm{s}} |c_m|^2 + \sum_{m=1}^{N_\mathrm{s}} \sum_{\substack{m=1,\\ m'\neq 1}}^{N_\mathrm{s}} c_{m'}^* c_m e^{\frac{i}{\hbar}\{\mathbf{p}_m - \mathbf{p}_{m'}\}\cdot\mathbf{x}}[u^s(p_{m'})]^\dagger[u^s(p_m)]\,, \tag{S3.2}$$

where the second summations contibute to shaping of the electron spatial probability distribution function. Similarly, we can rewrite the differential cross section expression from equation (S1.8) as

$$
\begin{aligned}
&\frac{\mathrm{d}\sigma}{\mathrm{d}\omega_{k'}\mathrm{d}\Omega_{k'}} \\
&= \sum_{r',s'} \left\{ \sum_{m=1}^{N_\mathrm{s}} |c_m|^2 \times \int \mathrm{d}\Omega_{p'} \delta_{E_p - \hbar\omega_{k'} - E_{p'}} \frac{\omega_{k'} |\mathbf{p}'|_0}{8\varepsilon_0 \{2\pi\}^5 \hbar^3 c^5 |\mathbf{p}|} \left| A_{\mathrm{Yu}}(\boldsymbol{\kappa}_m) \mathcal{M}_{k'p'p_m}^{r's's_m} \right|^2 \right. \\
&+ \sum_{m=1}^{N_\mathrm{s}} \sum_{\substack{m'=1, \\ m' \neq m}}^{N_\mathrm{s}} c_{m'}^* c_m \qquad \text{(S3.3)} \\
&\left. \times \int \mathrm{d}\Omega_{p'} \delta_{E_p - \hbar\omega_{k'} - E_{p'}} \frac{\omega_{k'} |\mathbf{p}'|_0}{8\varepsilon_0 \{2\pi\}^5 \hbar^3 c^5 |\mathbf{p}|} \left[A_{\mathrm{Yu}}(\boldsymbol{\kappa}_{m'}) \mathcal{M}_{k'p'p_{m'}}^{r's's_{m'}} \right]^* \left[A_{\mathrm{Yu}}(\boldsymbol{\kappa}_m) \mathcal{M}_{k'p'p_m}^{r's's_m} \right] \right\}
\end{aligned}
$$

As mentioned in the main text, the first summations can be referred as the incoherent component of the output emission (incoherent emission). The second summations can be seen as the interference component resulting from electron weaveshaping, and the overall summations give the coherent emission.

Incoherent summation of number of atoms N_a

We can fully expand the differential cross section from equation (S1.13) into summations of both number of electron momentum states N_s and number of atoms N_a as

$$
\begin{aligned}
\frac{\mathrm{d}\sigma}{\mathrm{d}\omega_{k'}\mathrm{d}\Omega_{k'}} = \sum_{r',s'} &\left\{ \sum_{n=1}^{N_\mathrm{a}} \sum_{n'=1}^{N_\mathrm{a}} \left[\sum_{m=1}^{N_\mathrm{s}} \sum_{m'=1}^{N_\mathrm{s}} c_{m'}^* c_m \right. \right. \\
&\times \int \mathrm{d}\Omega_{p'} \delta_{E_p - \hbar\omega_{k'} - E_{p'}} \frac{\omega_{k'} |\mathbf{p}'|_0}{8\varepsilon_0 \{2\pi\}^5 \hbar^3 c^5 |\mathbf{p}|} \qquad \text{(S3.4)} \\
&\left. \left. \times \left[a_{n'}(\boldsymbol{\kappa}_{m'}) a_n(\boldsymbol{\kappa}_m) e^{i(\boldsymbol{\kappa}_m \cdot \mathbf{d}_n - \boldsymbol{\kappa}_{m'} \cdot \mathbf{d}_{n'})} \right] \left[\mathcal{M}_{k'p'p_{m'}}^{r's's_{m'}} \right]^* \left[\mathcal{M}_{k'p'p_m}^{r's's_m} \right] \right] \right\}
\end{aligned}
$$

where Yukawa potential $a_n(\boldsymbol{\kappa}_m)$ is given in equation (S2.4) and $\boldsymbol{\kappa}_m = \frac{\mathbf{p}_m}{\hbar} - \mathbf{k}' - \frac{\mathbf{p}'}{\hbar}$. The term $a_{n'}(\boldsymbol{\kappa}_{m'}) a_n(\boldsymbol{\kappa}_m) e^{i(\boldsymbol{\kappa}_m \cdot \mathbf{d}_n - \boldsymbol{\kappa}_{m'} \cdot \mathbf{d}_{n'})}$ can be expressed in its Fourier form as

$$
\begin{aligned}
&a_{n'}(\boldsymbol{\kappa}_{m'})a_n(\boldsymbol{\kappa}_m)e^{i(\boldsymbol{\kappa}_m\cdot\mathbf{d}_n-\boldsymbol{\kappa}_{m'}\cdot\mathbf{d}_{n'})}\\
&=\left(\frac{q_e}{4\pi\varepsilon_0}\right)^2 Z_{n'}Z_n\sum_{j_n}\sum_{j'_{n'}}C_{j'_{n'}}C_{j_n}\\
&\times\int \mathrm{d}^3\mathbf{r}'\mathrm{d}^3\mathbf{r}\left[\frac{e^{-\frac{\mu_{j'_{n'}}}{a_0}|\mathbf{r}'-\mathbf{d}_{n'}|}e^{-i\left(\frac{\mathbf{p}_{m'}}{\hbar}-\mathbf{k}'\right)\cdot\mathbf{r}'}}{|\mathbf{r}'-\mathbf{d}_{n'}|}\right]\left[\frac{e^{-\frac{\mu_{j_n}}{a_0}|\mathbf{r}-\mathbf{d}_n|}e^{i\left(\frac{\mathbf{p}_m}{\hbar}-\mathbf{k}'\right)\cdot\mathbf{r}}}{|\mathbf{r}-\mathbf{d}_n|}\right]\left[e^{-i\frac{\mathbf{p}'}{\hbar}\cdot(\mathbf{r}-\mathbf{r}')}\right].
\end{aligned}
\tag{S3.5}
$$

Assume $\left[\mathcal{M}_{k'p'p_{m'}}^{r's's_{m'}}\right]^*\left[\mathcal{M}_{k'p'p_m}^{r's's_m}\right]$ are relatively slow varying in the integral of solid angle $\Omega_{p'}$, such that we can only focus on the integral $\int \mathrm{d}\Omega_{p'}\, e^{-i\frac{\mathbf{p}'}{\hbar}\cdot(\mathbf{r}-\mathbf{r}')}$ as

$$
\begin{aligned}
&\int \mathrm{d}^3\mathbf{r}'\mathrm{d}^3\mathbf{r}\left[\frac{e^{-\frac{\mu_{j'_{n'}}}{a_0}|\mathbf{r}'-\mathbf{d}_{n'}|}e^{-i\left(\frac{\mathbf{p}_{m'}}{\hbar}-\mathbf{k}'\right)\cdot\mathbf{r}'}}{|\mathbf{r}'-\mathbf{d}_{n'}|}\right]\left[\frac{e^{-\frac{\mu_{j_n}}{a_0}|\mathbf{r}-\mathbf{d}_n|}e^{i\left(\frac{\mathbf{p}_m}{\hbar}-\mathbf{k}'\right)\cdot\mathbf{r}}}{|\mathbf{r}-\mathbf{d}_n|}\right]\\
&\qquad\times\left[\int \mathrm{d}\Omega_{p'}e^{-i\frac{\mathbf{p}'}{\hbar}\cdot(\mathbf{r}-\mathbf{r}')}\right]\\
&\qquad=\int \mathrm{d}^3\mathbf{r}'\mathrm{d}^3\mathbf{r}\left[\frac{e^{-\frac{\mu_{j'_{n'}}}{a_0}|\mathbf{r}'-\mathbf{d}_{n'}|}e^{-i\left(\frac{\mathbf{p}_{m'}}{\hbar}-\mathbf{k}'\right)\cdot\mathbf{r}'}}{|\mathbf{r}'-\mathbf{d}_{n'}|}\right]\left[\frac{e^{-\frac{\mu_{j_n}}{a_0}|\mathbf{r}-\mathbf{d}_n|}e^{i\left(\frac{\mathbf{p}_m}{\hbar}-\mathbf{k}'\right)\cdot\mathbf{r}}}{|\mathbf{r}-\mathbf{d}_n|}\right]\\
&\qquad\times\left[4\pi\,\mathrm{sinc}\left[\frac{|\mathbf{p}'|}{\hbar}|\mathbf{r}-\mathbf{r}'|\right]\right].
\end{aligned}
\tag{S3.6}
$$

The magnitude of equation (S3.6) is determined by three fast decaying components (first two represent the Yukawa potentials, third is the sinc function), which peak at $\mathbf{r}'\approx\mathbf{d}_{n'}$, $\mathbf{r}\approx\mathbf{d}_n$ and $\frac{|\mathbf{p}'|}{\hbar}|\mathbf{r}-\mathbf{r}'|\approx 0$ respectively. Those three components only have their peaks overlap at $\mathbf{d}_{n'}=\mathbf{d}_n$, and thus those terms with $\mathbf{d}_{n'}=\mathbf{d}_n$ in equation (S3.4) are dominant, which gives

$$\frac{\mathrm{d}\sigma}{\mathrm{d}\omega_{k'}\mathrm{d}\Omega_{k'}} \approx \sum_{n=1}^{N_\mathrm{a}} \left\{ \sum_{m=1}^{N_\mathrm{s}} \sum_{m'=1}^{N_\mathrm{s}} c_{m'}^{*} c_m e^{i\left(\frac{\mathbf{p}_m}{\hbar} - \frac{\mathbf{p}_{m'}}{\hbar}\right)\cdot \mathbf{d}_n} \right.$$
$$\times \sum_{r',s'} \int \mathrm{d}\Omega_{p'}\, \delta_{E_p - \hbar\omega_{k'} - E_{p'}} \frac{\omega_{k'} |\mathbf{p}'|_0}{8\varepsilon_0 \{2\pi\}^5 \hbar^3 c^5 |\mathbf{p}|}$$
$$\left. \times \left[A_n(\boldsymbol{\kappa}_{m'}) \mathcal{M}_{k'p'p_{m'}}^{r's's_{m'}}\right]^{*} \left[A_n(\boldsymbol{\kappa}_m) \mathcal{M}_{k'p'p_m}^{r's's_m}\right] \right\} \tag{S3.7}$$
$$= \sum_{n=1}^{N_\mathrm{a}} \left\{ \sum_{r',s'} \int \mathrm{d}\Omega_{p'} \delta_{E_p - \hbar\omega_{k'} - E_{p'}} \frac{\omega_{k'} |\mathbf{p}'|_0}{8\varepsilon_0 \{2\pi\}^5 \hbar^3 c^5 |\mathbf{p}|} \right.$$
$$\left. \times \left| \sum_{m=1}^{N_\mathrm{s}} [A_n(\boldsymbol{\kappa}_m)] \left[c_m \mathcal{M}_{k'p'p_m}^{r's's_m}\right] \right|^2 \right\} .$$

Therefore, the total bremsstrahlung differential cross section can be treated as the incoherent summation of individual components occurring at each atom, hence results in the linear N_a scaling. This argument is valid in our interested regime, supported by our simulation results.

Paraxial approximation

To show that some of the results could be arrived at using the paraxial approximation, while others cannot, we assume identical spin $s_m = s$ for all m and

$$u^{s_m}(p_m) \approx u^s(p_0), \qquad A_\mathrm{Yu}(\boldsymbol{\kappa}_m) \approx A_\mathrm{Yu}(\boldsymbol{\kappa}_0), \qquad \mathcal{M}_{k'p'p_{m'}}^{r's's_m} \approx \mathcal{M}_{k'p'p_0}^{r's's}, \tag{S3.8}$$

where the subscript 0 denotes the unshaped electron momentum state, i.e., momentum solely in positive longitudinal direction (+z-axis). The paraxial approximation allows us to extract the integral component out of the summation over m in equation (S3.7), which gives

$$\left[\frac{\mathrm{d}\sigma}{\mathrm{d}\omega_{k'}\mathrm{d}\Omega_{k'}}\right]_{\mathrm{paraxial}}$$

$$\approx \sum_{n=1}^{N_{\mathrm{a}}}\left[\mathrm{P}_n \sum_{r',s'} \int \mathrm{d}\Omega_{p'} \delta_{E_p-\hbar\omega_{k'}-E_{p'}} \frac{\omega_{k'}|\mathbf{p}'|_0}{8\varepsilon_0\{2\pi\}^5\hbar^3 c^5|\mathbf{p}|}\left|A_n(\boldsymbol{\kappa}_0)\mathcal{M}_{k'p'p_0}^{r's's}\right|^2\right] \tag{S3.9}$$

where

$$\mathrm{P}_n = \sum_{m=1}^{N_{\mathrm{s}}}|c_m|^2 + \sum_{m=1}^{N_{\mathrm{s}}}\sum_{\substack{m'=1,\\ m'\neq m}}^{N_{\mathrm{s}}} c_{m'}^* c_m\, e^{i\left(\frac{\mathbf{p}_m}{\hbar}-\frac{\mathbf{p}_{m'}}{\hbar}\right)\cdot\mathbf{d}_n} \tag{S3.10}$$

can be interpreted as the electron spatial probability distribution function at n^{th} atom (refer to equation (S3.2)).

Conclusively speaking, the total bremsstrahlung differential cross section as the incoherent summation of individual components at each individual atom. Moreover, under the scheme of paraxial approximation, each component is directly proportional to the electron spatial probability distribution function at the corresponding atom location.

In Fig. 1, we show the scaling properties for shaped electron wavefunction converging to Bessel beam of order 0 (*off-axis emission*) and order 1 (*on-axis emission*). Since each electron momentum states with momentum $\mathbf{p}_m$ has their transverse momentum constructed from the reciprocal vectors of the lattice vectors $\mathbf{d}_n$ (in *xy*-plane), we have the relation $\frac{\mathbf{p}_m}{\hbar}\cdot\mathbf{d}_n = 2l\pi$ for all m and n, where $l \in \mathbb{Z}$. We can then calculate P_n for

(1) Discrete Bessel beam of order 0 *(off-axis emission*)

complex amplitude $c_m = \frac{1}{\sqrt{N_{\mathrm{s}}}}$ for all m, which gives

$$\mathrm{P}_n = \Psi_p^{\dagger}\Psi_p(\mathbf{d}_n) \approx \frac{1}{N_{\mathrm{s}}}\sum_{m=1}^{N_{\mathrm{s}}}\sum_{m'=1}^{N_{\mathrm{s}}} 1 = \frac{1}{N_{\mathrm{s}}}\times N_{\mathrm{s}}\times N_{\mathrm{s}} = N_{\mathrm{s}}\ ; \tag{S3.11}$$

(2) Discrete Bessel beam of order 1 (*on-axis emission*)

complex amplitude $c_m = \frac{1}{\sqrt{N_{\mathrm{s}}}}\mathrm{e}^{\mathrm{i}\{m-1\}\frac{2\pi}{N_{\mathrm{s}}}}$ for all m, which gives

$$\mathrm{Ƥ}_n = \Psi_p^\dagger \Psi_p(\mathbf{d}_n) \approx \frac{1}{N_\mathrm{s}} \sum_{m=1}^{N_\mathrm{s}} \sum_{m'=1}^{N_\mathrm{s}} \mathrm{e}^{\mathrm{i}\{m-m'\}\frac{2\pi}{N_\mathrm{s}}} = \frac{1}{N_\mathrm{s}} \frac{1-\mathrm{e}^{\mathrm{i}N_\mathrm{s}\frac{2\pi}{N_\mathrm{s}}}}{1-\mathrm{e}^{\mathrm{i}\frac{2\pi}{N_\mathrm{s}}}} \frac{1-\mathrm{e}^{-\mathrm{i}N_\mathrm{s}\frac{2\pi}{N_\mathrm{s}}}}{1-\mathrm{e}^{-\mathrm{i}\frac{2\pi}{N_\mathrm{s}}}} = 0 \ . \quad \text{(S3.12)}$$

For *off-axis emission* (equation (S3.11)), the paraxial approximation predicts linear N_s scaling $\left[\frac{\mathrm{d}\sigma}{\mathrm{d}\omega_{k'}\mathrm{d}\Omega_{k'}}\right]_{\mathrm{paraxial}} \propto N_\mathrm{s}$ which match our exact simulation. However, for *on-axis emission* (equation (S3.12)), the paraxial approximation predicts vanishing $\left[\frac{\mathrm{d}\sigma}{\mathrm{d}\omega_{k'}\mathrm{d}\Omega_{k'}}\right]_{\mathrm{paraxial}} \rightarrow 0$ (no emission), which is not the case from our exact simulation. These examples show that paraxial approximation does not work universally for all the cases of shaped electron wavefunction, which outlines the non-intuitive features of electron waveshaping in QED process.

Non-recoil approximation

For extremely small photon energy $\hbar\omega_{k'}$ (compared to input electron kinetic energy E_p), i.e. $\hbar\omega_{k'} \ll E_p$, one can further approximate the supposing energy conservation Dirac delta $\delta[E_p - \hbar\omega_{k'} - E_{p'}] \approx \delta[E_p - E_{p'}]$, i.e. electron energy is treated as $E_{p'} \approx E_p$ for negligible recoil.

The results implemented with both paraxial and non-recoil approximations are compared with the exact simulation in Fig. 3 from the main text. The full paraxial and non-recoil approximation still predict the linear N_s scaling for *off-axis emission*, but the scaling factor differ from exact simulation due to large emitted photon energy (large recoil).

<u>Section 4 | Further examples of tailoring bremsstrahlung via electron waveshaping</u>

As a counterpart for Fig. 4, we show examples in Fig. S4 with a different set of parameters, i.e., a much smaller incidence angle (0.005π rad) for all the electron momentum states. One can observe that the angular emission profile of the bremsstrahlung inherits the symmetries of the shaped electron wavefunction (in xy-plane).

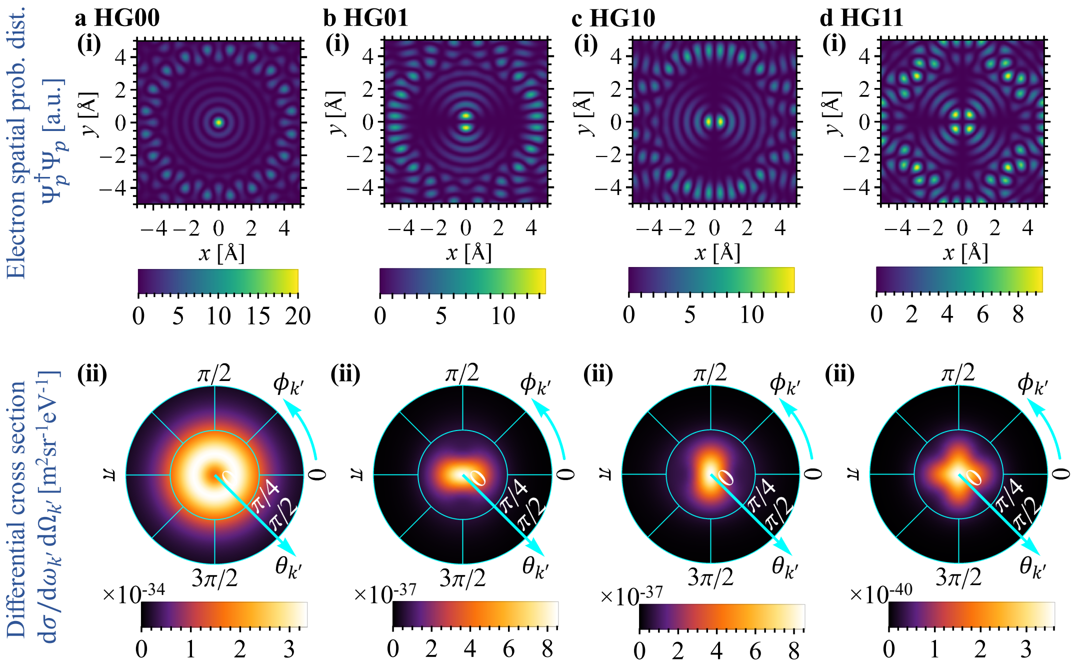

Fig. S4 Complex shaping of bremsstrahlung emission via engineering multi-state electron wavefunctions. Panels **a - d(i)** show the spatial probability distribution (prob. dist.) of 20-state 300 keV electron (incident angle 0.005π rad) converging to **a(i)** HG00 beam, **b(i)** HG01 beam, **c(i)** HG10 beam and **d(i)** HG11 beam, respectively. Panels **a - d(ii)** show the corresponding bremsstrahlung differential cross section $d\sigma/d\omega_{k'}d\Omega_{k'}$ (photon energy 250 keV).

Section 5 | Enhancement of bremsstrahlung X-ray in 3D bulk crystalline materials

In this section, we extend our theory to 3D bulk crystalline materials, i.e., multilayer graphite. In Fig. S5, we show that the bremsstrahluhnf X-ray differential cross section scales linearly as the number of layers of graphite, for both unshaped (1-state) and shaped (6-state) electron wavepackets, which the later also shows a 6-times enhancement. Using 6-state shaped electron wavepacket, we are able to make the electron's periodic spatial profile to be focused at each carbon atoms of the graphite, as shown in the inset of Fig. S5. The shaped electron's spatial profile remains invariant along the longitudinal direction (z-axis), resulting in the same contribution to the emitted X-ray photon flux from each layers of the graphite crystal plane.

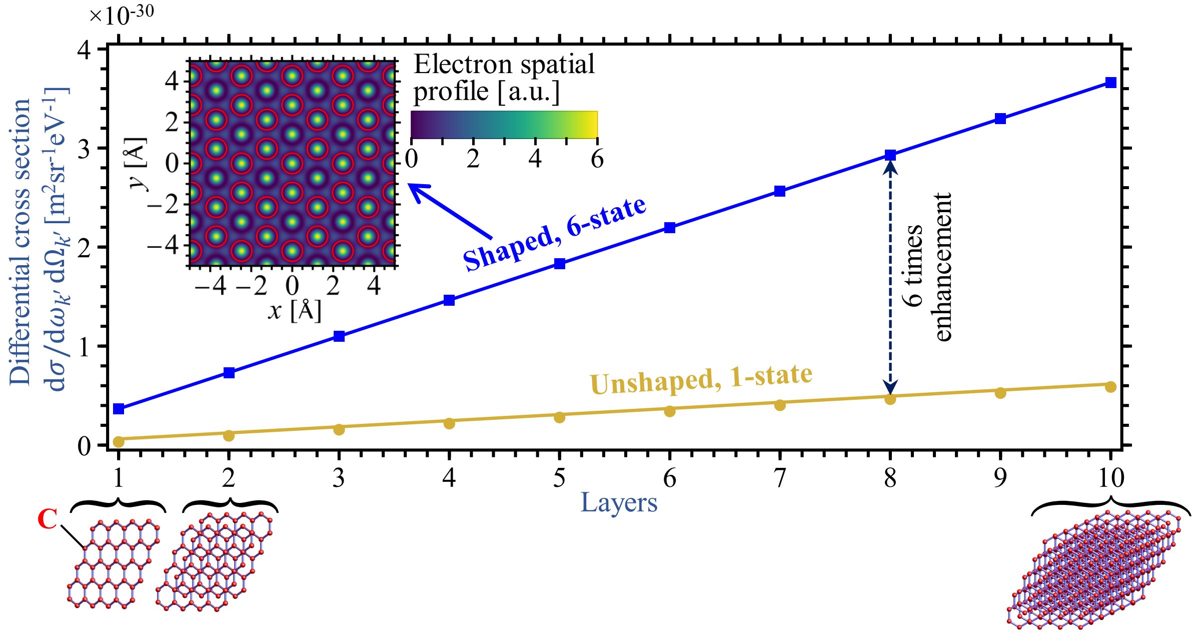

Fig. S5 Linear enhancement of bremsstrahlung X-ray in multilayer graphite. In both cases of unshaped (1-state) and shaped (6-state) electron wavepackets, the resulting bremsstrahlung X-ray differential cross sections (at optimum emission angles $\theta_{k'}$= 0.3 [π rad], $\phi_{k'}$= 0 [π rad]) scale linearly as the number of layers of graphite. Each layer consists of 48 C atoms. The inset depicts the 6-state shaped electron's spatial profile (in xy-plane), which is periodically aligned with the C atoms (denoted by red circles) and remains invariant along z-direction. Compared to unshaped case, the case of 6-state shaped electron also shows a 6-fold enhancement in the X-ray differential cross section.

Section 6 | Experimental proposals for proof-of-concept demonstrations

The purpose of this section is to propose experiments that can demonstrate the main conclusions of this work, and also to show that the needed experimental apparatus are commercially available or well-within the state-of-the-art. The setup we propose is shown in Fig. S6, and can be realized, for instance, in a customized transmission electron microscope (TEM). The electron source is a field emission gun (FEG) or photoemission DC gun that produces 20 to 300 keV electron beam with high spatial coherence, similar to those used in electron holography. The electron's quantum wavefunction is then shaped by passing the electron through an adaptive electrostatic phase plate with 36 programmable elements, a realistic device given that 48-element programmable phase plates have been very recently designed and demonstrated [5]. Our proposed experiments require the phase plate's elements to possess phase resolution of $1\times10^{-2}\ \pi$, which is well within reach today given that the aforementioned 48-element phase plate has been reported to possess phase resolution of $3\times10^{-3}\ \pi$ [5]. New electron waveshaping techniques, such as using short intense laser pulses [6], surface plasmon polaritons [7] etc. to modulate the electron beam are being developed, and can potentially be utilized in future experiments. The shaped electron beam then impinges on the 2D crystal, which can be chosen from the enormous family of 2D van der Waals materials. In our experimental plan presented here, we choose graphene as an exemplary target material for generating bremsstrahlung. The single-layer graphene is mounted on a nanopositioning piezoelectric stage. To ensure that the shaped electron's transverse spatial profile is aligned to the crystal lattice periodicity and stabilized, we require the stage to possess the following properties: (1) sub-nanometer mechanical resolution, e.g., < 0.02 nm, as has been realized in ref. [8], for closed loop control (feedback in real time); (2) 6 degrees of freedom, i.e., 3 linear and 3 rotational motion axes, as has been realized in ref. [9]; and (3) a clear aperture that allows transmission of X-rays [9]. As these specifications are well-within the state-of-the-art and even realized in existing commercial products, such a nanopositioning piezoelectric stage is feasible today, at the expense of some customization in the worst case scenario. The generated X-ray photon is captured by an X-ray detector. The X-ray detector is mounted on a motorized stage, which can be rotated to

enable a sufficient portion of the X-ray emission angular profile to be recorded and compared against the results of our theoretical predictions.

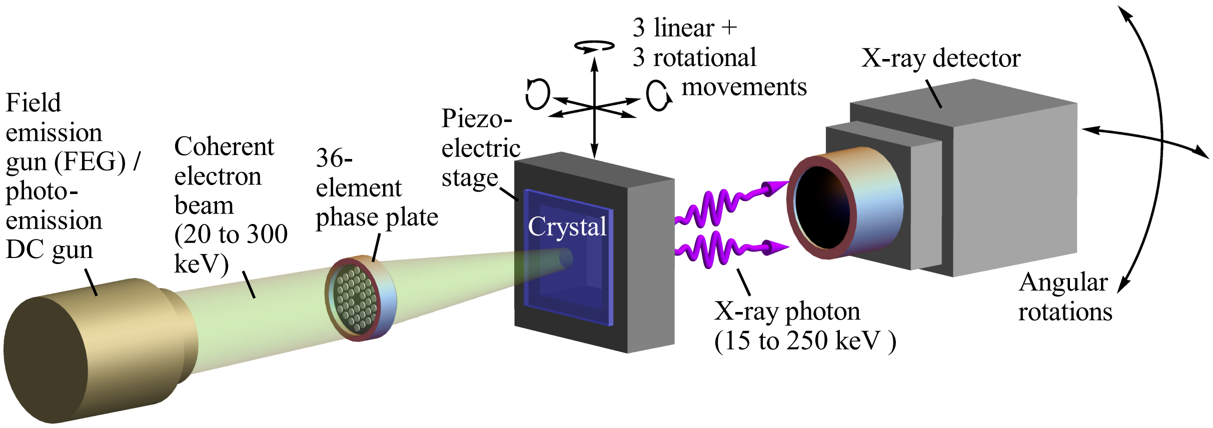

Fig. S6 Proposed experimental setup for demonstrating enhancement and shaping of bremsstrahlung by electron waveshaping. The field emission gun (FEG) or photoemission DC gun produces 20 to 300 keV electron beam with high spatial coherence. A 36-element programmable phase plate is used to shape the electron transverse profile into periodic patterns matching the crystal lattice. A nanopositioning piezoelectric stage with 3 linear (resolution < 0.02 nm) and 3 rotational (resolution < 0.1 μrad) motion axes controls the alignment between electron transverse profile and crystal lattice with real time feedback (closed loop control). Bremsstrahlung X-ray in forward direction is generated when the electron impinges the crystal, transmitted through the clear aperture of the stage, and recorded by the X-ray detector. The X-ray detector can move angularly to fully cover the emission angular profile of the X-ray.

Using the above proposed experimental setup, we design two experiments that would provide proof-of-concept confirmation for our theory, while achieving unprecedented enhancement and shaping of X-ray bremsstrahlung. The aim of the first proposed experiment (Fig. S7) is to demonstrate the enhancement of bremsstrahlung -- we show in Fig. S7 that our theory predicts as much as 18 times intensity enhancement. The aim of the second proposed experiment (Fig. S8) is to demonstrate the ability to shape the bremsstrahlung angular profile by shaping the electron wavefunction -- we show in Fig. S8 that our theory predicts that the emission pattern can indeed be substantially altered using experimental parameters that are already feasible today. Our results not only serve as a confirmation that the phenomena we predict are within reach of experimental setups that are realistic today, but further motivates the already rapidly progressing development of multi-element phase plates for electron waveshaping and high-precision nanopositioning stages.

We now delve into the technical details surrounding our two proposed experiments. In the experimental plan illustrated in Fig. S7, we use 300 keV electron source and 2D graphene as the cyrstalline scatterer for bremsstrahlung. For the first experiment, we investigate the enhancement in output bremsstrahlung X-ray photon flux via electron waveshaping. We use the 36-element phase plate (illustrated in Fig. S7 a(i), 50 μm in diameter [5]) to split the electron beam into 36 components. Each components is converged into different incident angles, which corresponds to a specific electron momentum state. Under ideal conditions, the resulting waveshaped electron spatial profile (in *xy*-plane, $z = 0$) is shown in Fig. S7 a(ii), aligned to the graphene lattice structure (red circles representing carbon atoms). Our theory predicts that an enhancement of around 18 times for 250 keV X-ray output flux (Fig S7 b(ii)) compared to unshape case (Fig S7 b(i)) would be experimentally observed.

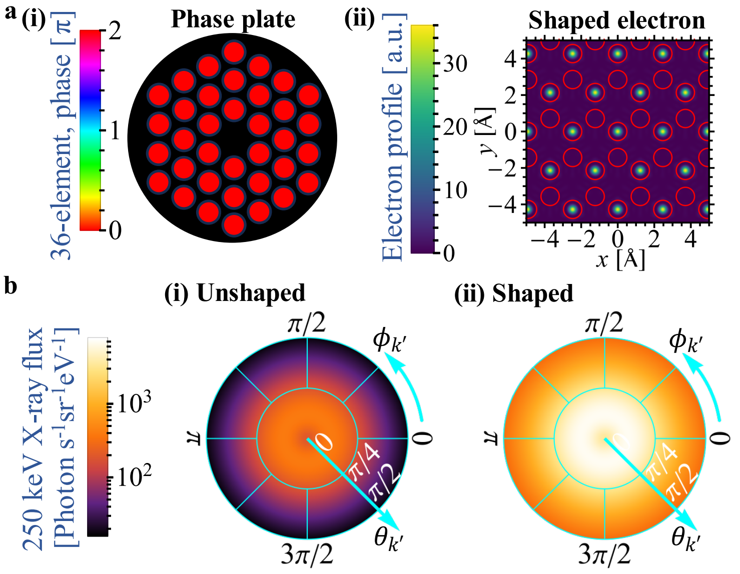

Fig. S7 Proposed experiment to demonstrate enhancement of bremsstrahlung X-ray flux intensity up to 18 times via electron waveshaping. We assume 300 keV electron beam of current density 10^7 A/m^2 and an active area of 100 μm^2 (the area which the electron periodic spatial profile is considered ideally aligned with the graphene atoms). In a(i), the 36-element phase plate shapes the electron beam into 36-state beam, each momentum state has incident angle corresponding to the position of each phase element, i.e., 9.21 mrad, 15.95 mrad, 18.42 mrad, 24.37 mrad and 27.64 mrad, from inner to outer position. Panel a(ii) shows the shaped electron periodic spatial profile, ideally aligned with the graphene lattice where the C atoms are denoted by red circles. The 250 keV output X-ray flux emission angular profiles for unshaped and shaped cases are simulated and presented in b(i) and b(ii), respectively, showing enhancement up to 18 times in X-ray flux intensity for waveshaped electron.

In the experimental plan depicted in Fig. S8, we explore the ability of electron waveshaping to tailor the bremsstrahlung X-ray emission angular profile. Using the same 36-element phase plate, we vary the phases of each splitted components that corresponds to a specific electron momentum state, as shown in Figs. S8 a(i) and b(i), respectively. Under ideal conditions, the resulting waveshaped electron spatial profiles (in xy-plane, $z = 0$) are shown in Figs. S8 a(ii) and b(ii) respectively, aligned to the graphene lattice structure (red circles representing carbon atoms). The corresponding simulated emission angular profiles for 250 keV X-ray are presented in Figs. S8 a(iii) and b(iii), respectively, showing distinct shapes that would provide a strong proof-of-concept for the shaping of the bremsstrahlung emission angular profile via electron waveshaping.

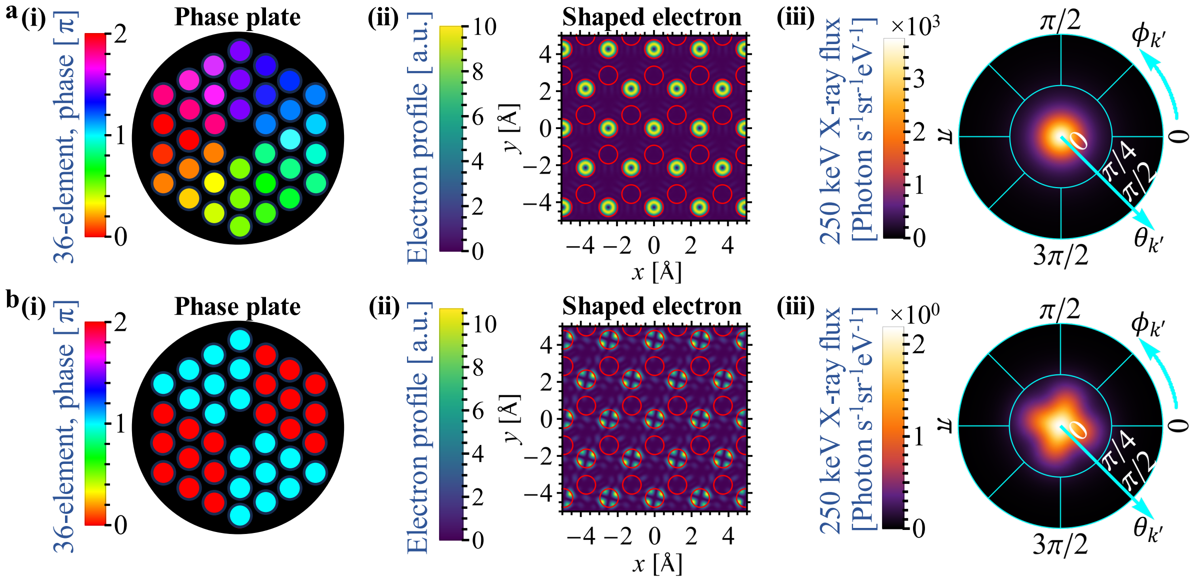

Fig. S8 Proposed experiment to demonstrate shaping of the X-ray emission angular profile by shaping the electron wavefunction. We assume 300 keV electron beam of current density 10^9 A/m^2 and an active area of 100 μm^2 (the area which the electron periodic spatial profile is considered ideally aligned with the graphene atoms). In a(i) and b(i), we assume two phase configurations for the 36-element phase plate in order to obtain distinct electron waveshaping results. Panels a(ii) and b(ii) show the corresponding shaped electron periodic spatial profiles, ideally aligned with the graphene lattice (C atoms are denoted by red circles). The output 250 keV X-ray flux emission angular profile for both shaped cases are simulated and presented in a(iii) and b(iii), respectively, which the former shows a uniform central peak and the latter shows a 4-pointed-star shape.

References

[1] Peskin, M. E. & Schroeder, D. V. *An Introduction to Quantum Field Theory* (Westview, 1995).

[2] Salvat, F., Martinez, J. D., Mayol, R. & Parellada, J. Analytical Dirac–Hartree–Fock–Slater screening function for atoms (Z = 1–92*). Phys. Rev. A* **36**, 467 (1987).

[3] Salvat, F. Elastic scattering of fast electrons and positrons by atoms. *Phys. Rev. A* **43**, 578 (1991).

[4] Jain, A. *et al.* Commentary: The Materials Project: A materials genome approach to accelerating materials innovation. *APL Materials* **1**, 1 (2013).

[5] Yu, C.-P. *et al.* Quantum Wavefront Shaping with a 48-element Programmable Phase Plate for Electrons. arXiv:2308.16304 (2023).

[6] Chirita Mihaila, M. C. *et al.* Transverse Electron-Beam Shaping with Light. *Physical Review X* **12**, 031043 (2022).

[7] Tsesses, S. *et al.* Tunable photon-induced spatial modulation of free electrons. *Nature Materials* **22**, 345–352 (2023).

[8] Nano-HS Series | High Speed, Compact, XY and XYZ Nanopositioning Systems with Picometer Resolution for AFM, SPM, and Metrology. http://www.madcitylabs.com/nanohsseries.html (2008).

[9] Physik, I. 562.6CD PIMars 6-Axis Nanopositioning Stage. https://www.pi-usa.us/en/products/piezo-flexure-nanopositioners/6-axis-piezo-nanopositioning-stages/p-5626cd-pimars-6-axis-nanopositioning-stage-201555 (2021).
